# Astrocytes expressing mutant SOD1 and TDP43 trigger motoneuron death that is mediated via sodium channels and nitroxidative stress

**DOI:** 10.3389/fncel.2014.00024

**Published:** 2014-02-07

**Authors:** Fabiola Rojas, Nicole Cortes, Sebastian Abarzua, Agnieszka Dyrda, Brigitte van Zundert

**Affiliations:** Faculty of Biological Sciences and Faculty of Medicine, Center for Biomedical Research, Universidad Andres BelloSantiago, Chile

**Keywords:** ALS, non-cell-autonomous, motor neuron, degeneration, ROS/RNS, anti-oxidants

## Abstract

Amyotrophic lateral sclerosis (ALS) is a fatal paralytic disorder caused by dysfunction and degeneration of motor neurons. Multiple disease-causing mutations, including in the genes for SOD1 and TDP-43, have been identified in ALS. Astrocytes expressing mutant SOD1 are strongly implicated in the pathogenesis of ALS: we have shown that media conditioned by astrocytes carrying mutant SOD1^G93A^ contains toxic factor(s) that kill motoneurons by activating voltage-sensitive sodium (Na_*v*_) channels. In contrast, a recent study suggests that astrocytes expressing mutated TDP43 contribute to ALS pathology, but do so via cell-autonomous processes and lack non-cell-autonomous toxicity. Here we investigate whether astrocytes that express diverse ALS-causing mutations release toxic factor(s) that induce motoneuron death, and if so, whether they do so via a common pathogenic pathway. We exposed primary cultures of wild-type spinal cord cells to conditioned medium derived from astrocytes (ACM) that express SOD1 (ACM-SOD1^G93A^ and ACM-SOD1^G86R^) or TDP43 (ACM-TDP43^A315T^) mutants; we show that such exposure rapidly (within 30–60 min) increases dichlorofluorescein (DCF) fluorescence (indicative of nitroxidative stress) and leads to extensive motoneuron-specific death within a few days. Co-application of the diverse ACMs with anti-oxidants Trolox or esculetin (but not with resveratrol) strongly improves motoneuron survival. We also find that co-incubation of the cultures in the ACMs with Na_*v*_ channel blockers (including mexiletine, spermidine, or riluzole) prevents both intracellular nitroxidative stress and motoneuron death. Together, our data document that two completely unrelated ALS models lead to the death of motoneuron via non-cell-autonomous processes, and show that astrocytes expressing mutations in SOD1 and TDP43 trigger such cell death through a common pathogenic pathway that involves nitroxidative stress, induced at least in part by Na_*v*_ channel activity.

## Introduction

Amyotrophic lateral sclerosis (ALS) is a fatal paralytic disorder caused by the progressive degeneration of cranial and spinal motoneurons in adulthood, and leading to death by respiratory failure within 3–5 years of diagnosis. Although the majority of ALS cases are sporadic (SALS), ~10% are familial (FALS) and are generated by mutations in at least 15 identified ALS-associated gene loci (Bento-Abreu et al., 2010; Ferraiuolo et al., 2011). Dominant mutations in superoxide dismutase 1 (SOD1) and transactive response DNA-binding protein 43 (TARDBP gene, TDP43 protein) are common causes of ALS (Cleveland and Rothstein, 2001; Pasinelli and Brown, 2006; Cozzolino et al., 2012)—to date, more than 150 SOD1 mutations and 40 TARDBP mutations are known to be associated with the ALS phenotype (http://alsod.iop.kcl.ac.uk/; Abel et al., 2012).

Although the molecular underpinnings of motoneuron degeneration in ALS have not yet been elucidated, *in vivo* and *in vitro* studies with use of transgenic mice that carry ALS-causing mutants reveal a large number of pathogenic changes in affected motoneurons: these include mitochondrial dysfunction, hyperexcitability, glutamate excitotoxicity, nitroxidative stress from reactive oxygen species (ROS) or reactive nitrogen species (RNS) (collectively leading to nitroxidative stress), protein aggregation and misfolding, proteasome impairment, cytoskeletal disruption, activation of cell death signals, and dysregulation of transcription and RNA processing (Beckman et al., 2001; Cleveland and Rothstein, 2001; Bruijn et al., 2004; Pasinelli and Brown, 2006; Ferraiuolo et al., 2011; Cozzolino et al., 2012; van Zundert et al., 2012). Despite these advances in identifying these cellular alterations, however, the origin(s) and interplay between multiple pathogenic processes of motoneuron death in ALS remain largely unknown.

A large number of studies highlight the importance of dysregulated crosstalk between motoneurons and non-neuronal cells in ALS (Ilieva et al., 2009). The notion that ALS is at least partially a non-cell-autonomous disease originates in a groundbreaking study from Clement et al. (2003) who generated chimeric mice composed of mixtures of normal and SOD1 mutant-expressing cells, and showed that wild-type non-neuronal cells extend the survival of motoneurons carrying mutant SOD1. Additional research has since firmly established the contribution of “deadly neighboring cells” (astrocytes, microglia, oligodendrocytes, and Schwann cells) to the degeneration of motoneurons (Boillée et al., 2006; Yamanaka et al., 2008a,b; Lobsiger et al., 2009; Ilieva et al., 2009). Other findings offer compelling evidence that primary mutant SOD1-expressing astrocytes from mouse (Di Giorgio et al., 2007; Nagai et al., 2007; Castillo et al., 2013; Fritz et al., 2013), rat (Vargas et al., 2006; Cassina et al., 2008), and humans (Marchetto et al., 2008) effectively and selectively kill motoneurons, but spare interneurons. Importantly, astrocytes differentiated from neuronal progenitor cells (NPCs) obtained either from post-mortem spinal cord tissue or from skin biopsies from FALS (SOD1 mutations and hexanucleotide expansion in C9orf72) and SALS patients also display non-cell-autonomous toxicity, and selectively kill motoneurons in a co-culture model system (Haidet-Phillips et al., 2011; Meyer et al., 2014). Moreover, astrocytes that express mutants in SOD1 contribute to the pathogenesis of ALS by releasing into the media a toxic factor(s) that kills motoneurons (Nagai et al., 2007; Cassina et al., 2008; Castillo et al., 2013; Fritz et al., 2013). Little is known regarding the non-cell- autonomous toxicity mediated by mutants other than in SOD1, but a recent study suggests that astrocytes expressing mutated TDP43 (TDP43^M337V^) lack non-cell-autonomous toxicity and contribute to ALS pathology only through cell-autonomous processes (Serio et al., 2013).

Here we show that conditioned medium derived from astrocytes that were harvested from transgenic mice carrying ALS-causing mutations in SOD1 (SOD1^G93A^ and SOD1^G86R^) or TDP43 (TDP43^A315T^) contain toxic factors that trigger motoneuron death. Based on earlier studies which document the involvement of Na_*v*_ channel-mediated excitability and nitroxidative stress in the pathogenesis of ALS (Ferraiuolo et al., 2011; Cozzolino et al., 2012; van Zundert et al., 2012), we tested here whether these pathogenic changes are induced in motoneurons via non-cell-autonomous processes. We demonstrate that they do so, and our results indicate that nitroxidative stress within the neurons is mediated by Na_*v*_ channel activity.

## Materials and methods

### Animals

Care and use of rodents was in accordance with the US National Institute of Health guidelines, and was approved by the Institutional Animal Care and Use Committee of Andres Bello University. Hemizygous transgenic mice carrying mutant human SOD1^G93A^ (high copy number; B6SJL; Cat. No. 002726), wild-type human SOD1^WT^ (B6SJL; Cat. No. 002297), mutant mouse SOD1^G86R^ (FVB crossed on B6SJL background; Cat. No. 005110), or mutant mouse TDP43^A315T^ (B6.Cg crossed on C57BL/6J; Cat. No. 010700) were originally obtained from Jackson Laboratories (Bar Harbor, USA). Non-transgenic littermates and transgenic mice over-expressing the gene for human SOD1^WT^ were used as controls. Transgenes were identified by polymerase chain reaction (Wegorzewska et al., 2009; Castillo et al., 2013; Fritz et al., 2013). The SOD1^G93A^ mice, but not the hSOD1^WT^ mice, develop signs of neuromuscular deficits (tremor of the legs and loss of extension reflex of the hind paws) starting at 3 months of age and have an average lifespan of 19–21 weeks (Gurney et al., 1994). Mice carrying SOD1^G86R^ (Ripps et al., 1995) or TDP43^A315T^ (Wegorzewska et al., 2009) develop similar loss of motor function between 3 and 4 months and do not survive to the age of 4 months.

### Conditioned media preparation

ACM was prepared as described (Nagai et al., 2007; Castillo et al., 2013; Fritz et al., 2013). Briefly, cultures of astrocytes were prepared from P1-2 wild-type mice and from transgenic mice expressing human SOD1^G93A^, mouse hSOD1^G86R^, human SOD1^WT^, or mouse TDP43^A315T^. Cultures were maintained in DMEM (Hyclone, Cat. No. SH30081.02) containing 10% FBS (Hyclone, Cat. No. SH30071.03; lot ATC31648) and 1% penicillin-streptomycin (Gibco, Cat. No. 15070-063) at 37°C 5% CO_2_. Cultures reached confluence after 2–3 weeks and contained >95% GFAP^+^ astrocytes. Residual microglia were removed by shaking cultures in an orbital shaker (200 r.p.m. in the incubator) overnight (7 h), at which point media was replaced by spinal culture media (see below). After 7 days, ACM was collected, centrifuged (500 g for 10 min) and stored at −80°C; before use, it was supplemented with 4.5 mg/ml D-glucose (final concentration) and penicillin/streptomycin, and filtered. A chick hindlimb muscle extract was also added to the ACM before use (Sepulveda et al., 2010).

For all experiments the ACM was diluted 8–10-fold. The exact dilution was determined for each new batch of ACM by comparing the motoneuron toxicity of the ACM from transgenic animals carrying the ALS-causing mutants (ACM-SOD1^G93A^, ACM-SOD1^G86R^, and ACM-TDP43^A315T^) to that of ACM generated from mice carrying the wild-type human SOD1 gene (ACM-SOD1^WT^) or from non-transgenic littermates (ACM-NT-Control); at the selected dilutions the conditioned media derived from the astrocytes expressing the ALS-causing genes robustly killed motoneurons, whereas the ACM-NT-Control and ACM-SOD1^WT^ did not affect motoneuron survival. The ACM was applied to ventral spinal cord cultures derived from rats because better quality motoneurons are obtained from rats than from mice; a number of studies have shown that such mixed species co-cultures (from rat, mice, human) do not appear to induce any side effects (e.g., Pehar et al., 2004; Di Giorgio et al., 2007; Nagai et al., 2007; Castillo et al., 2013; Fritz et al., 2013).

### Primary spinal cord neuronal cultures

Pregnant Sprague–Dawley rats were deeply anesthetized with CO_2_, and primary spinal cultures were prepared from E14 pups (Sepulveda et al., 2010; Castillo et al., 2013; Fritz et al., 2013). Briefly, whole spinal cords were excised and placed into ice-cold HBSS (Gibco, Cat. No. 14185-052) containing 50 μg/ml penicillin/streptomycin (Gibco, Cat. No. 15070-063). The dorsal part of the spinal cord was removed using a small razor blade, and the ventral cord was minced and enzymatically treated by incubating in pre-warmed HBSS containing 0.25% trypsin (Gibco, Cat. No. 15090-046) for 20 min at 37°C. Cells were transferred to a 15 ml tube containing neuronal growth media containing 70% MEM (Gibco, Cat. No. 11090-073), 25% Neurobasal media (Gibco, Cat. No. 21103-049), 1% N2 supplement (Gibco, Cat. No. 17502-048), 1% L-glutamine (Gibco, Cat. No. 25030-081), 1% penicillin-streptomycin (Gibco, Cat. No. 15070-063), 2% horse serum (Hyclone, Cat. No. SH30074.03; lot AQH24495) and 100 mM sodium pyruvate (Gibco, Cat. No. 11360-070); they were precipitated, transferred to a new 15-ml-tube containing 2 ml of growth media, re-suspended by mechanical agitation through fire-polished glass Pasteur pipettes of different tip diameters, and counted; 1 × 10^6^ cells were plated on freshly prepared poly-L-lysine-coated 24-well plates (1 mg/ml; 30.000–70.000 mW; Sigma, Cat. No. P2636). Cells were cultured for 7 days at 37°C under 5% CO_2_, and supplemented with 45 μg/ml chick hindlimb muscle extract (Sepulveda et al., 2010); the media was refreshed every 3 days.

### Pharmacological treatments in culture

Mexiletine (Tocris, Cat. No. 2596) was dissolved in water to 100 mM and used at final concentration of 25 nM. Riluzole (Sigma, Cat. No. R116) was dissolved in distilled water (plus 10% Tween20) at 100 μM, and added to cultures to final concentration of 100 nM. Spermidine (Sigma, Cat. No. S2626) was dissolved in water at 100 mg/ml and added to cultures to a final concentration of 10 μM. Trolox (Sigma, Cat. No. 238813) was dissolved in distilled water at 100 mM and added to cultures to final concentration of 1 μM. Esculetin (Sigma, Cat. No. 17795) was dissolved in dimethyl sulfoxide (DMSO), and added to cultures to final concentration 25 μM. Resveratrol (Tocris, Cat. No. 1418) was dissolved in DMSO (Sigma) at 100 mM and added to cultures to final concentration of 1 μM. All stock solutions were stored at −20°C.

### Cell labeling and counting

Motoneurons and interneurons were immunolabeled and counted as previously described (Sepulveda et al., 2010; Castillo et al., 2013; Fritz et al., 2013). Briefly, primary spinal cultures were fixed at 7 DIV with 4% paraformaldehyde, and immunostained with an antibody against MAP2 (1:400; Santa Cruz Biotechnology) to label all neurons (interneurons plus motoneurons) and with the SMI-32 antibody (1:1,000, Sternberger Monoclonals) to reveal the presence of unphosphorylated neurofilament-H, which is expressed specifically in motoneurons in spinal cord cultures (Urushitani et al., 2006; Nagai et al., 2007); antibody binding was visualized with the appropriate fluorescent secondary antibodies. Our wild-type primary spinal cord cultures typically contain at least 6–10% motoneurons until 12 DIV (Sepulveda et al., 2010). Immunolabeled neurons were documented on an inverted Nikon Eclipse Ti-U microscope equipped with a SPOT Pursuit™ USB CameraCCD (14-bit), Epi-fl Illuminator, mercury lamp, and Sutter Smart-Shutter with a lambda SC controller. Cells were photographed using a 20× objective; MAP2- and SMI-32-positive neurons were counted offline within 20 randomly chosen fields, and the percentage of SMI-32-positive motoneurons within the total number of MAP2-positive cells was calculated. Each condition was replicated in at least 3 independent cultures, and in duplicate.

### Nitroxidative stress measurements with CM-H_2_DCF-DA

The intracellular levels of ROS/RNS were measured with CM-H_2_DCF-DA (Invitrogen, Cat. No. C6827). H_2_DCF-DA is not a specific probe for a particular oxidant and has been used to monitor certain ROS/RNS (see Discussion). The CM-H_2_DCF-DA stock solution (5 mM) was prepared in DMSO and was diluted in the culture medium to a final concentration of 1 μ M just before addition to the cells. After application of the diverse ACMs to the spinal cord cultures for different time (minutes-hours-days), cells were washed (PBS 1×) to remove the ACMs and exposed to CM-H_2_DCF-DA for 30 min at 37°C in dark, to label both motoneurons and interneurons. To facilitate the CM-H_2_DCF-DA membrane penetration, 0.004% Pluronic acid F-127 (Invitrogen, Cat. No. P-3000MP) was added to the culture medium to facilitate dye entry, eliminating possible hydrolysis of dyes by external esterases and maintain better cell integrity (Appaix et al., 2012). After the incubation time, the CM-H_2_DCF-DA-cointaing culture medium was removed and cultures were washed twice with PBS 1× and suspended in culture medium (500 μ l final volume). Next, cells were immediately imaged using an inverted Nikon Eclipse Ti-U microscope equipped with a SPOT Pursuit™ USB CameraCCD (14-bit), Epi-fl Illuminator, mercury lamp, and Sutter Smart-Shutter with a lambda SC controller. Cells were photographed using a 20× objective. As CM-H_2_DCF-DA is a non-fluorescent dye it passively diffuses into cells and is hydrolyzed intracellularly to the DCFH carboxylate anion that is trapped inside; oxidation of DCFH results in the formation of the fluorescent product DCF, with excitation and emission wavelengths λ_ex_/λ_em_ = 492–495/517–527 nm. The exposure time was kept below 4 s in order to avoid photo-oxidation of the ROS/RNS sensitive dye and for all given treatments fields were exposed for exactly the same amount of time. At least three independent fields were acquired for each condition and at least 10 cells per field were used for quantification of the fluorescence signal. Cells were marked by drawing a region of interest around the cell body, and mean fluorescence intensity was calculated for each cell after subtraction of the background signal using the image analysis module in ImageJ software. Those cells with a relative intensity unit (RIU) of ≥1.5 were counted as positive. Cultures were also incubated with H_2_O_2_ (200 μM for 20 min) to serve as a positive control and to normalize the number of DCF-positive cells after ACM application.

### Data analysis

ANOVA, followed by *post-hoc* Tukey tests, was used to detect significant changes. Student's *t-*tests were used to compare the response of two cell populations to individual treatments. Unless otherwise stated, error bars represent the mean ± s.e.m.; ^*^p < 0.05, ^**^p < 0.01, ^***^p < 0.001 vs. control.

## Results

### ACM-SOD1^G93A^, ACM-SOD1^G86R^, or ACM-TDP43^A315T^ triggers death of cultured primary motoneurons

**Figure 1:**
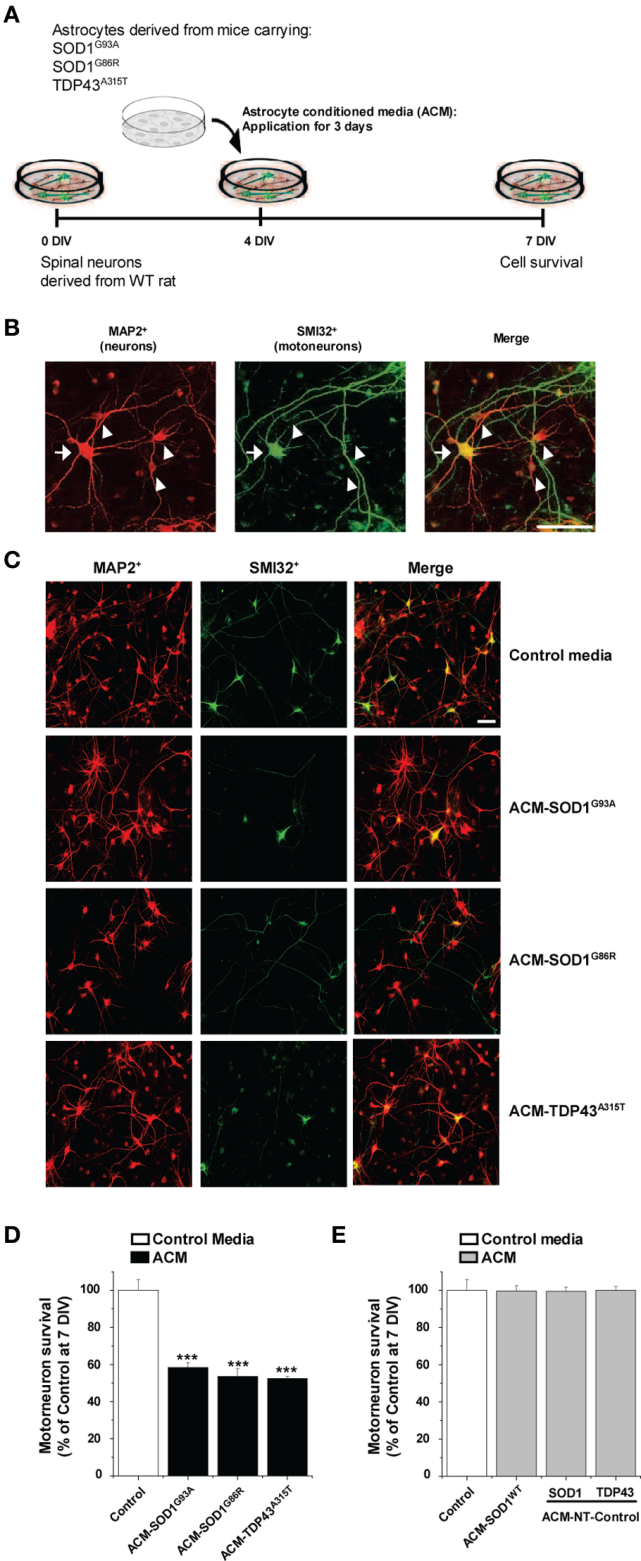
**Exposure of primary spinal cord cultures to astrocyte conditioned media (ACM) derived from SOD1^G93A^, SOD1^G86R^, and TDP43^A315T^ expressing mice triggers death of motoneurons. (A)** Flow diagram of experiment. Primary wild-type (WT) rat spinal cord cultures (4 DIV) were exposed for 3 days to ACM derived from transgenic mice overexpressing SOD1^G93A^ (ACM-SOD1^G93A^), SOD1^G86R^ (ACM-SOD1^G86R^), or TDP43^A315T^ (ACM-TDP43^A315T^). Cells were fixed at 7 DIV, and cell survival was assayed with immunocytochemistry. **(B)** Fixed 7 DIV spinal cord cultures were double-labeled with anti-microtubule-associated protein 2 (MAP2) antibody (red) to visualize interneurons (arrowhead) and with the SMI-32 antibody (green) to identify motoneurons (arrow). Scale bar, 25 μm. **(C)** Representative images of MAP2^+^/SMI32^+^-labeled neurons in spinal cultures under control conditions (top image) or treated with the three different ACMs, as indicated in the figure. Scale bar, 200 μm. **(D)** Graph showing the percentage of motoneurons that survived after treatment with ACMs derived from SOD1^G93A^, SOD1^G86R^, and TDP43^A315T^ astrocytes, relative to motoneurons from sister cultures treated with control medium. **(E)** Graph showing the percentage of motoneurons that survived after treatment with media derived from mouse littermates that were negative for the mutated forms of SOD1 and TDP43 (ACM-NT-Control), or with media derived from transgenic mice carrying the non-pathological human wild-type SOD1 gene (ACM-SOD1^WT^). Survival is shown relative to cultures treated with control media. Values represent mean ± s.e.m. from at least 3 independent experiments performed in duplicate, analyzed by One-Way ANOVA followed by a Tukey *post-hoc* test. ^***^P < 0.001 relative to control medium at 7 DIV.

Here we investigated whether astrocytes expressing diverse ALS causing mutants release toxic factor(s) that induce motoneuron death, and if so, whether a common pathogenic pathway is involved. Astrocyte conditioned media (ACM) was derived from astrocytes that were harvested from mice carrying mutant SOD1 (ACM-SOD1^G93A^ and ACM-SOD1^G86R^) or TDP43 (ACM-TDP43^A315T^). These media were added at 8–10-fold dilutions (see Materials and Methods) to wild-type primary rat spinal cultures at 4 DIV for 3 days; effects on neuron survival were assessed at 7 DIV (Figure 1A). To define the presence of all neurons, an antibody against microtubule-associated protein 2 (MAP2) was used (Figure 1B; arrowhead); motoneurons were specifically identified with use of the SMI-32 antibody, which recognizes unphosphorylated neurofilament-H (Figure 1B; arrow), as previously described (Urushitani et al., 2006; Nagai et al., 2007; Sepulveda et al., 2010; Castillo et al., 2013; Fritz et al., 2013). Chronic (3 days) exposure (from 4 to 7 DIV) of spinal cultures to ACM-SOD1^G93A^, ACM-SOD1^G86R^, or ACM-TDP43^A315T^ induced ~50% motoneuron death (Figures 1C,D). The number of interneurons was unchanged after application of ACM-SOD1^G93A^ (91 ± 1% vs. control, *p* > 0.05 by *t*-test), ACM-SOD1^G86R^ (96 ± 19% vs. control, *p* > 0.05 by *t*-test), or ACM-TDP43^A315T^ (103 ± 23% vs. control, *p* > 0.05 by *t*-test). Our findings that ACM-SOD1^G93A^ and ACM-SOD1^G86R^ robustly reduces motoneuron cell survival, while sparing interneurons, are consistent with previous studies (Nagai et al., 2007; Castillo et al., 2013; Fritz et al., 2013). Our results with ACM-TDP43^A315T^ also show for the first time that astrocytes carrying a TDP43 mutant kill motoneurons through non-cell-autonomous processes.

Three types of control media were used throughout this work. (1) “Control” media that was not conditioned by astrocytes. (2) “ACM-NT-Control” media derived from astrocytes that were harvested from littermate mice that were negative for the SOD1 and TDP43 gene. (3) “ACM-SOD1^WT^” media derived from astrocytes that were harvested from transgenic mice carrying the non-pathological human wild-type SOD1 gene. None of these media caused motoneuron death (Figure 1E). In particular, the finding that ACM-SOD1^WT^ was not toxic indicates that the factor inducing motoneuron death is specifically due to the SOD1^G93A^ or SOD1^G86R^ mutation, rather than to overexpression of the human SOD1 protein. By contrast, we can not exclude the possibility that motoneuron death in our spinal cultures is attributable, at least in part, by increased levels of the TDP43 protein itself. In fact, accumulating data with transgenic models have established that excessive levels of even human wild-type TDP43 result in neurodegeneration, likely as a result in the disruption of RNA metabolism (Wegorzewska et al., 2009; Wils et al., 2010; Igaz et al., 2011; Ling et al., 2013).

### ACM-SOD1^G93A^, ACM-SOD1^G86R^, or ACM-TDP43^A315T^ leads to increases in intracellular ROS/RNS levels

**Figure 2:**
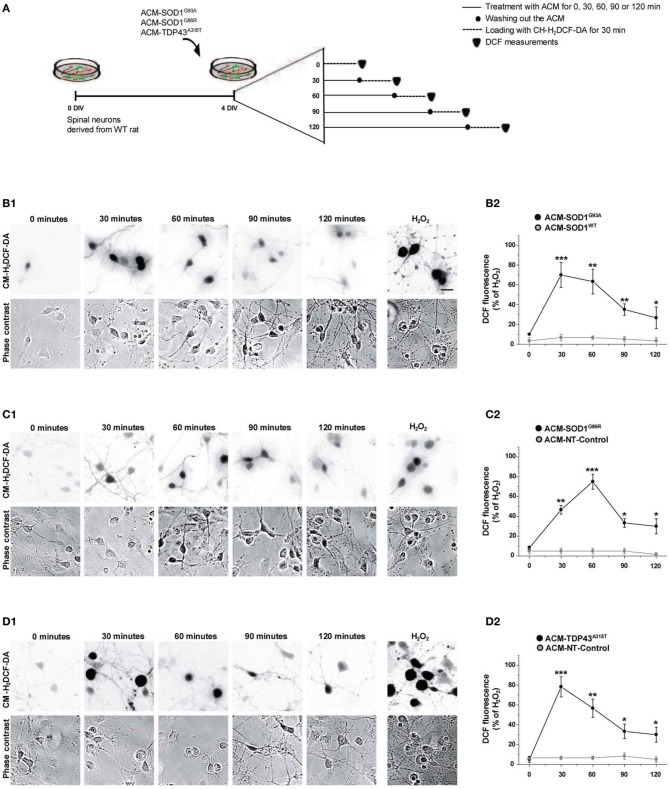
**Exposure of primary spinal cord cultures to ACM-SOD1^G93A^, ACM-SOD1^G86R^, and ACM-TDP43^A315T^ induces rapid increases in intracellular dichlorofluorescein (DCF) fluorescence. (A)** Flow diagram of experiment. Primary wild-type spinal cultures (4 DIV) were exposed for 0–120 min with the different ACMs (solid lines), washed to remove the ACMs (filled circles), and loaded for 30 min with the fluorescent membrane permeable ROS/RNS probe CM-H_2_DCF-DA (dotted lines). Next cultures were washed and DCF measurements were immediately performed (filled triangles). **(B–D)** Cultures exposed to ACM-SOD1^G93A^ **(B)**, ACM-SOD1^G86R^ **(C),** or ACM-TDP43^A315T^ **(D)**. **(B**_1_–**D**_1_**(D)** The negatives of representative DCF fluorescent images (in which both motoneurons and interneurons are marked) and corresponding phase contrast images of spinal cord cultures photographed at 0, 30, 90 and 120 min after application of ACM-SOD1^G93A^ **(B**_1_**(D)**, ACM-SOD1^G86R^ **(C**_1_**)**, or ACM-TDP43^A315T^ **(D**_1_**)** are shown. In all experiment, H_2_O_2_ (200 μM for 20 min) served as positive control and to normalize the number of DCF-positive cells after ACM application. Scale bar, 200 μm. **(B**_2_–**D**_2_**)** Graphs showing the percentage of DCF fluorescent cells (including both motoneurons and interneurons) under the conditions indicated. Results obtained with different controls are included in the graphs: control media, ACM-NT-Control, and ACM-SOD1^WT^. DCF fluorescence is relative to cultures treated with H_2_0_2_ (100%). Note that DCF fluorescence peaked after 30 min of incubation of spinal cultures with ACM-SOD1^G93A^ and ACM-TDP43^A315T^ **(B**_2_,**D**_2_**)**, and after 60 min for incubation with ACM-SOD1^G86R^ **(C**_2_**)**. Values represent mean ± s.e.m. from at least 3 independent experiments performed in duplicate, analyzed by *t*-test. ^*^p < 0.05, ^**^p < 0.01, ^***^p < 0.001 vs. control.

Increased intracellular levels of nitroxidative stress are widely and consistently observed in ALS patients, and in *in vitro* and *in vivo* mouse models that express SOD1 mutants (Barber and Shaw, 2010). To investigate whether soluble toxic factors released by astrocytes that carry SOD1 and TDP43 mutants induce an oxidative burden in primary neurons, we exposed 4 DIV cultures to the diverse ACMs for 30–120 min, washed cells to remove the ACMs, and subsequently loaded the cells with CM-H_2_DCF-DA for 30 min (Figure 2A). CM-H_2_DCF-DA is a non-fluorescent dye that passively diffuses into cells and is hydrolyzed intracellularly to the DCFH carboxylate anion that is trapped inside; oxidation of DCFH results in the formation of the fluorescent product DCF. Increased intensity in fluorescent DCF could thus reflect detection of certain reactive oxygen and nitrogen species, inducing nitroxidative stress. Combined real-time fluorescence and phase-contrast imaging showed that DCF levels were very low under basal culture conditions, while application of ACM-SOD1^G93A^ induced both a gradual increase in the intensity and the number of neurons displaying intracellular DCF fluorescence in spinal cord cultures; the fluorescence reached a peak at 30 min (Figure 2B). This increase was mimicked by H_2_O_2_ (Figure 2B; 200 μM for 20 min) but was blocked by use of diverse general anti-oxidants (see Figure 4). In control experiments, application of ACM-SOD1^WT^ did not change DCF fluorescence over the same exposure times (Figure 2B_2_). Exposure of spinal cord cultures to either ACM-SOD1^G86R^ (Figure 2C) or ACM-TDP43^A315T^ (Figure 2D) also triggered a gradual increase of intracellular DCF fluorescence in neurons; this fluorescence peaked at 60 and 30 min, respectively. Treatment of neuronal cultures with either ACM-NT-Control (Figures 2C_2_–D_2_) or control media did not induce significant differences (see Supplementary Figure 1 for images of all controls). Together, these results indicate that toxic factors released by astrocytes that carry diverse ALS-causing mutants results in increased nitroxidative stress in spinal cord neurons. The increased nitroxidative stress, however, is unable to induce immediate cell death as the number of motoneurons only starts to significantly reduce after 24 h of ACM application (Supplementary Figure 2).

### Anti-oxidants prevent motoneuron death induced by ACM-SOD1^G93A^, ACM-SOD1^G86R^, or ACM-TDP43^A315T^

**Figure 3:**
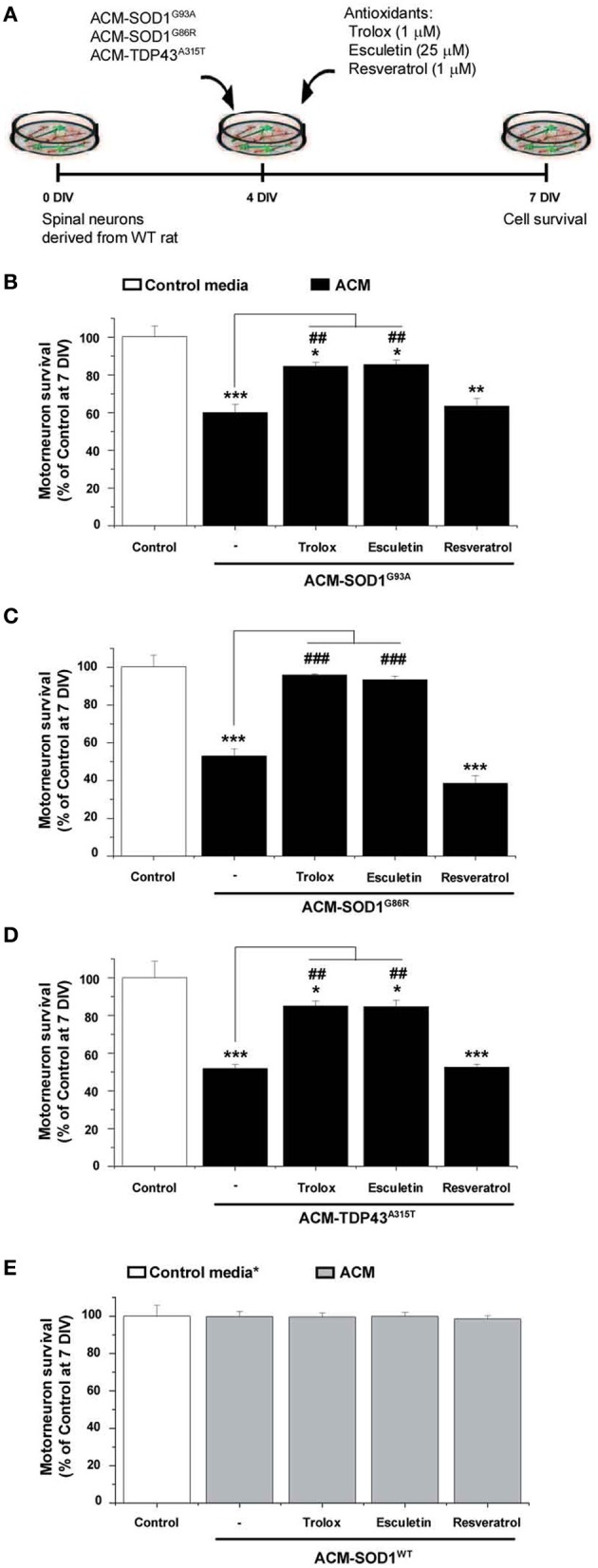
**Anti-oxidants Trolox and esculetin prevent motoneurons death induced by ACM-SOD1^G93A^, ACM-SOD1^G86R^, and ACM-TDP43^A315T^. (A)** Flow diagram of experiment. ACMs were applied chronically starting at 4 DIV alone, or together with the anti-oxidants Trolox (1 μ M), esculetin (25 μ M), or resveratrol (1 μ M). Cell survival was assayed at 7 DIV. **(B–E)** Graphs showing the relative percentage of motoneurons that survived at 7 DIV, after being treated with the diverse anti-oxidants and ACM-SOD1^G93A^ **(B)**, ACM-SOD1^G86R^ **(C)**, ACM-TDP43^A315T^ **(D)**, or ACM-SOD^WT^ **(E)**, relative to motoneurons from sister cultures treated with control medium (indicated with^*^) or with only the ACM (indicated with^#^). Note that Trolox and esculetin prevented motoneuron death induced by the diverse ALS-causing ACMs, while resveratrol was ineffective. Note also that none of the compounds improved the survival of control neurons. Values represent means ± s.e.m. from at least 3 independent experiments, analyzed by One-Way ANOVA followed by a Tukey *post-hoc* test. ^*^P < 0.05, ^**^P < 0.01, ^***^P < 0.001 relative to survival with control media at 7 DIV; ^##^*P* < 0.01 and ^###^*P* < 0.001 compared to survival with ALS-causing ACM at 7 DIV. See Supplementary Table 1. for the effects of a wide range of concentrations of anti-oxidants on motoneuron survival.

**Figure 4:**
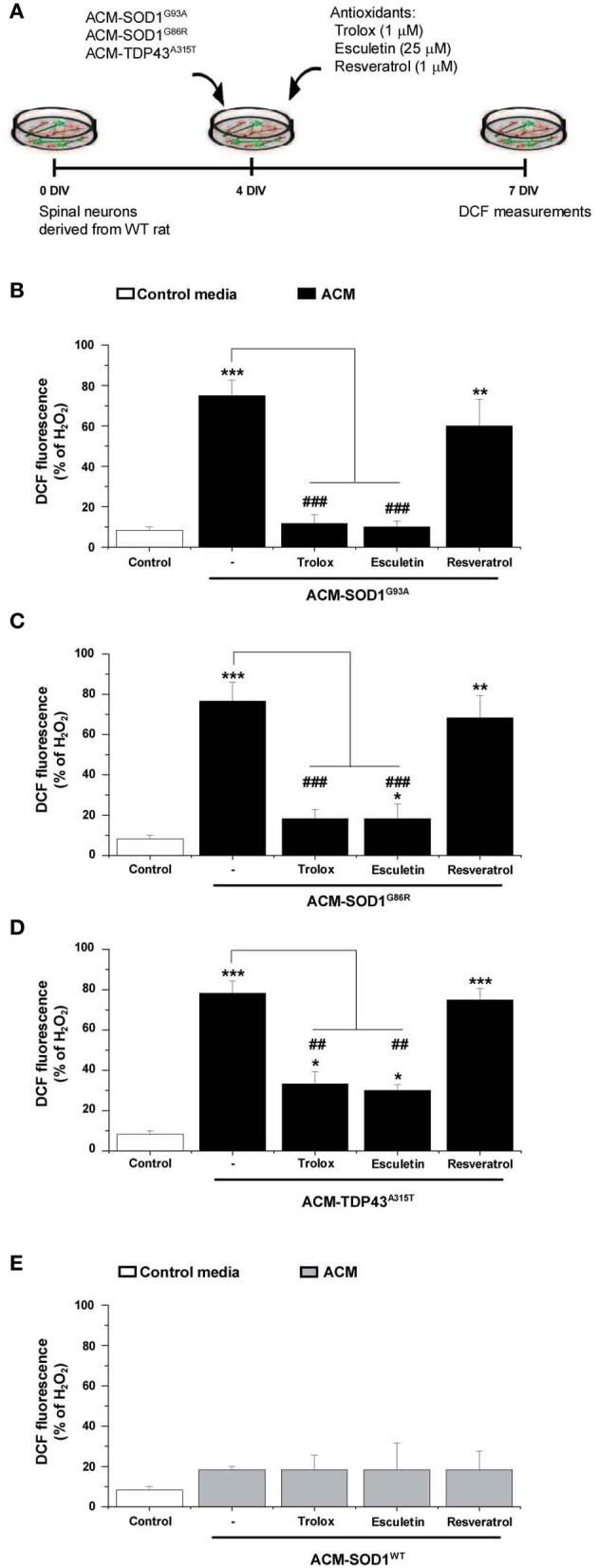
**The effect of anti-oxidants on DCF fluorescence in spinal cord cultures exposed to the diverse ACMs. (A)** Flow diagram of experiment. ACMs were applied chronically starting at 4 DIV alone, or together with the anti-oxidants Trolox (1 μ M), esculetin (25 μ M), or resveratrol (1 μ M). At 7 DIV, cultures were incubated with the membrane permeable ROS/RNS probe CM-H_2_DCF-DA and DCF fluorescence was measured 30 min later. **(B–E)** Graphs showing the percentage of DCF fluorescent cells after being treated with the diverse anti-oxidants and ACM-SOD1^G93A^ **(B)**, ACM-SOD1^G86R^ **(C)**, ACM-TDP43^A315T^ **(D)**, or ACM-SOD^WT^ **(E)**, In all experiment, H_2_O_2_ (200 μM for 20 min) served as positive control and to normalize the number of DCF-positive cells after ACM application. The graphs indicate statistics relative to motoneurons from sister cultures treated with control medium (indicated with^*^) or with only the ACM (indicated with^#^). Note that co-application of the diverse ACMs with Trolox or esculetin resulted in significant lower DCF fluorescent intensities, whereas resveratrol was not effective. Values represent means ± s.e.m. from at least 3 independent experiments, analyzed by One-Way ANOVA followed by a Tukey *post-hoc* test. ^**^P < 0.01, ^***^P < 0.001 relative to DCF fluorescence with control media at 7 DIV; ^##^*P* < 0.01 and ^###^*P* < 0.001 compared to DCF fluorescence with ALS-causing ACM at 7 DIV.

To determine whether the increased nitroxidative stress induced by the diverse ACMs contributes to motoneuron cell death, 4 DIV spinal cord neurons were chronically incubated in a combination of the toxic media plus one of the following anti-oxidants: Trolox, esculetin or resveratrol (Figure 3A). These anti-oxidants are reported to reduce intracellular levels of ROS/RNS (also documented in the present study—see Figure 4 for effects on DCF fluorescence; also see reference citations below). Multiple doses (ranging from 100 nM to 100 μM) of the anti-oxidants were used to assess the survival of motoneurons under control conditions, and after co-application with ACM-SOD1^G93A^ (see Supplementary Table 1). For each anti-oxidant drug, the maximum effect in preventing motoneuron death induced by ACM-SOD1^G93A^ is displayed in Figure 3B; the same concentrations of anti-oxidants were also used to test whether they could rescue motoneuron death induced by ACM-SOD1^G86R^ (Figure 3C) or ACM-TDP43^A315T^ (Figure 3D) (see below for details on each anti-oxidant). We also applied these doses of anti-oxidants to cultures incubated with ACM-SOD1^WT^ and found that none of the anti-oxidants were successful in significantly increasing the number of motoneurons (Figure 3E); similar results were obtained when anti-oxidants were co-applied to spinal cord culture with control media or ACM-NT-Control (not shown), indicating that the beneficial effects of these compounds are specifically attributable to counterbalancing increased nitroxidative stress induced by the diverse toxic ACMs, rather than to simply increasing overall motoneuron cell survival in the cultures.

We first analyzed use of vitamin E, the most potent naturally occurring scavenger of reactive oxygen and nitrogen species known (Tucker and Townsend, 2005). Extensive studies in ALS patients and mice models have shown, however, that vitamin E application *in vivo* is not capable of significantly prolonging survival (Gurney et al., 1996; Desnuelle et al., 2001; Ascherio et al., 2005; Graf et al., 2005) These disappointing results are likely related to the findings that vitamin E poorly penetrates the blood-brain barrier, leading to insufficient doses of this anti-oxidant in the central nervous system; IC_50_ of vitamin E is between 1.5 and 59 μM while ventricular CSF concentration of this vitamin was found at 0.114 μM (reviewed in Barber and Shaw, 2010). Here we opted to use Trolox, a water-soluble vitamin E analog that neutralizes ROS (Ghiselli et al., 1995; Khaldy et al., 2000; Barber et al., 2009; Distelmaier et al., 2012). Chronic co-application of ACM-SOD1^G93A^ (Figure 3B) or ACM-TDP43^A315T^ (Figure 3D) with 1 μM of Trolox significantly improved motoneuron survival. In the spinal cord cultures treated with ACM-SOD1^G86R^ plus Trolox improvement of motoneurons survival was even better and comparable with motoneuron survival obtained under control conditions (Figure 3C).

Next we analyzed esculetin and resveratrol, two molecules that have anti-oxidant activities (Lin et al., 2000; Kaneko et al., 2003; Baur and Sinclair, 2006; Barber et al., 2009). Esculetin and resveratrol were also identified from a screen of the Spectrum Collection library (consisting of 2000 small compounds) as two of the best-hit molecules, based on their ability to function as effective anti-oxidants by reducing DCF fluorescence and to increase the viability of a mutant SOD1^G93A^-expressing cell line; moreover, *in silico* analysis predicted that these two compounds have specific biochemical properties that allow efficient blood-brain barrier penetration (Barber et al., 2009). We found that chronic co-application of ACM-SOD1^G93A^ (Figure 3B) or ACM-TDP43^A315T^ (Figure 3D) with 25 μM esculetin significantly improved the survival of motoneurons and, in fact, treatment of spinal cord neurons with esculetin plus ACM-SOD1^G86R^ enhanced motoneuron survival to the level obtained under control conditions (Figure 3C).

By contrast, chronic application of resveratrol at 1 μM (Figure 3B)—as well as at a wide range of concentrations (100 nM to 10 μM) (Supplementary Table 1)—failed to prevent motoneuron death induced by ACM-SOD1^G93A^. Co-application of 1 μM resveratrol also did not significantly improve survival of motoneurons incubated with either ACM-SOD1^G86R^ (Figure 3C) or ACM-TDP43^A315T^ (Figure 3D). This negative effect was not related to toxicity of this anti-oxidant, as overall motoneuron cell survival in cultures treated with resveratrol was similar to that achieved under control conditions or cultures treated with ACM-SOD1^WT^ (see Figure 3E and Supplementary Table 1).

As discussed before, the application of the diverse ACMs to spinal cord cultures resulted in a strong DCF signals at 30–60 min which then gradually reduced (Figure 2). However, this is only a transient reduction and cells start to steadily increase DCF fluorescence from 24 h after ACM application; 7 DIV neurons treated chronically for 3 days with the diverse ACMs resulted in robust levels of ROS/RNS (~70–80% DCF fluorescence relative to H_2_O_2_) (Supplementary Figure 3). We next analyzed whether the doses of anti-oxidants that effectively increased cell survival (as shown in Figure 3) also prevented the increase in ROS/RNS levels induced by the diverse ACMs (Figure 4A). Co-application of ACM-SOD1^G93A^ plus Trolox (1 μM) or eculetin (25 μM) to spinal cord cultures decreased intracellular DCF fluorescence to a degree similar to that achieved in control cultures (Figure 4B). By contrast, resveratrol (1 μM) slightly, but not significantly, reduced nitroxidative stress induced by ACM-SOD1^G93A^. We also observed that co-application of Trolox and esculetin with ACM-SOD1^G86R^ (Figure 4C) or ACM-TDP43^A315T^ (Figure 4D) had similar beneficial effects in preventing nitroxidative stress; however, again, resveratrol was not effective in significantly reducing the DCF signal. This negative effect of resveratrol was not due to the absence of its anti-oxidant capacity; resveratrol reduced DCF fluorescence induced by H_2_O_2_ (200 μM for 20 min) by ~50% (Supplementary Figure 4).

Application of these anti-oxidants to control cultures (not shown), or to spinal cord cultures treated with either ACM-NT-Control (not shown) or with ACM-SOD1^WT^ (Figure 4E) revealed that none of these compounds led to significant decreases in basal DCF fluorescence intensity. Together, our data indicate that the favorable effects of Trolox and esculetin on motoneuron survival principally result from counterbalancing the increases in intracellular levels of ROS induced in neurons by the toxic actions of ACM-SOD1^G93A^, ACM-SOD1^G86R^, or ACM-TDP43^A315T^.

### Na_*v*_ channel blockers rescue motoneuron death induced by ACM-SOD1^G93A^, or ACM-TDP43^A315T^

**Figure 5:**
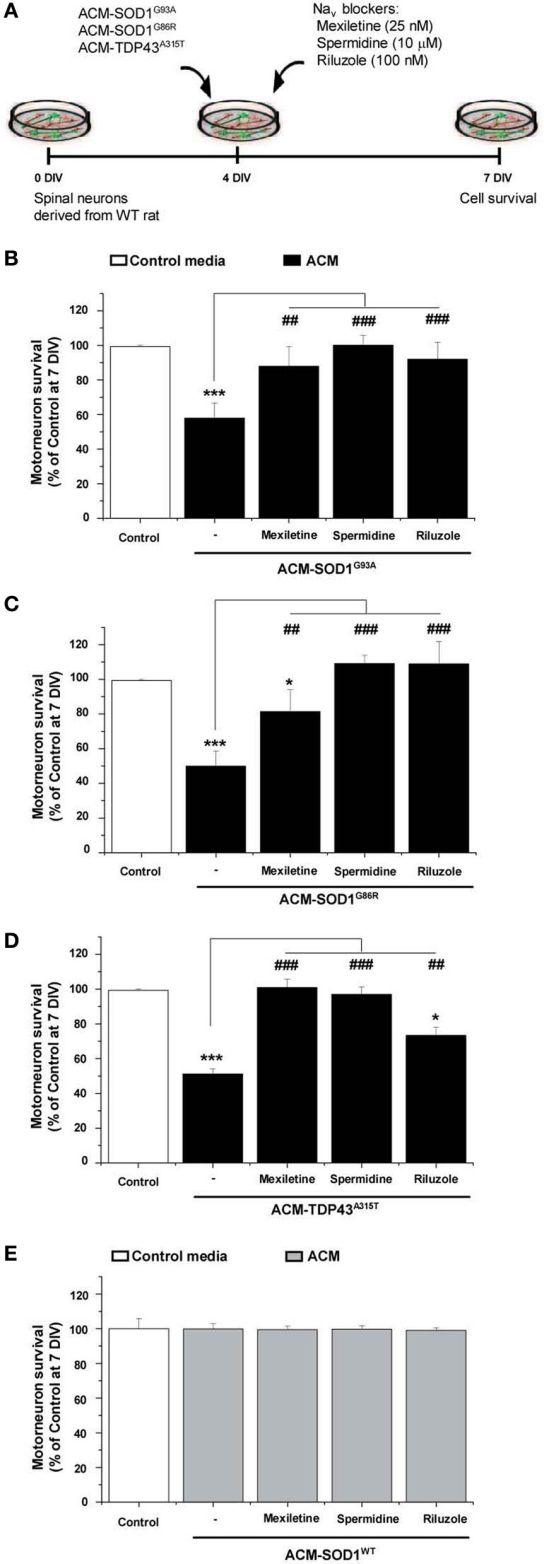
**Na_*v*_ channel blockers prevent motoneuron death induced by ACM-SOD1^G93A^, ACM-SOD1^G86R^, and ACM-TDP43^A315T^. (A)** Flow diagram of experiment. ACMs were applied chronically starting at 4 DIV alone, or together with the Na_*v*_ channel blockers mexiletine (25 nM), spermidine (10 μ M), or riluzole (100 nM). Cell survival was assayed at 7 DIV. **(B–E)** Graphs showing the relative percentage of motoneurons that survived at 7 DIV, after being treated with the diverse Na_*v*_ channel blockers and ACM-SOD1^G93A^ **(B)**, ACM-SOD1^G86R^ **(C)**, ACM-TDP43^A315T^ **(D)**, or ACM-SOD1^WT^ **(E)**, relative to motoneurons from sister cultures treated with control medium (indicated with^*^) or with only the ACM (indicated with^#^). Values represent means ± s.e.m. from at least 3 independent experiments, analyzed by One-Way ANOVA followed by a Tukey *post-hoc* test. ^*^P < 0.05, ^***^P < 0.001 relative to survival with control media at 7 DIV; ^##^*P* < 0.01 and ^###^*P* < 0.001 compared to survival with ALS-causing ACM at 7 DIV.

To gain insights into the mechanism whereby astrocytes expressing diverse ALS-causing mutant proteins increase intracellular ROS/RNS levels and kill motoneurons, we argued that if the conditioned media from the SOD1 and TDP43 mutant astrocytes share a toxic factor(s), then this toxicity must converge to a common target. We recently reported that ACM-SOD1^G93A^ rapidly (within 30 min) increases neuronal Na_*v*_ channel mediated excitability; moreover, the application of several blockers of the Na_*v*_ channel activity (including use of mexiletine, spermidine, and riluzole) reduced the hyperexcitability and prevented motoneuron death induced by ACM-SOD1^G93A^ (Fritz et al., 2013). Hence we tested whether the Na_*v*_ channel blocker mexiletine (an orally active lidocaine analog that is a local anesthetic and an antiarrhytmic drug that targets the “local anesthetic receptor site” of Na_*v*_ channels (Ragsdale et al., 1994; Catterall et al., 2005; Olschewski et al., 2009), spermidine (a polyamine that affects the gating of varies ion channels and serves as an endogenous, activity-dependent Na_*v*_ channel blocker (Williams, 1997; Fleidervish et al., 2008) and riluzole which has multiple effects, but at low concentrations (e.g., 100 nM in spinal cord cultures) suppresses neuronal excitability by affecting Na_*v*_ channels (Kuo et al., 2005; Theiss et al., 2007; Bellingham, 2011; Fritz et al., 2013) can also rescue motoneuron cell death induced by ACM-SOD1^G86R^ or ACM-TDP43^A315T^ (Figure 5A). We used the same concentrations of these Na_*v*_ channel blockers as described previously: 25 nM mexiletine, 10 μM spermidine and 100 nM riluzole (Fritz et al., 2013)—these doses were chosen based on earlier determinations that at these concentrations the Na_*v*_ channel blockers reduced excitability and also effectively prevented motoneuron cell death induced by ACM-SOD1^G93A^, without affecting overall motoneuron cell survival in control cultures (Fritz et al., 2013; and see also Figures 5B,E).

To directly compare the beneficial effect of co-application of mexiletine, spermidine or riluzole on the diverse toxic ACMs, we first analyzed motoneuronal survival when these sodium channel blockers were co-applied with ACM-SOD1^G93A^ (Figure 5B). As expected, chronic application (from 4 to 7 DIV) of mexiletine together with ACM-SOD1^G93A^ to spinal cord cultures prevented motoneuron death in those cultures (Figure 5B), as we have shown earlier (Fritz et al., 2013). Addition of mexiletine to spinal cord cultures exposed to ACM-SOD1^G86R^ (Figure 5C) and ACM-TDP43^A315T^ (Figure 5D) was also very beneficial and resulted in partially or complete prevention of motoneuron cell death, respectively. Spermidine (10 μM) also completely rescued motoneuron from death induced by ACM-SOD1^G93A^ (Figure 5B), ACM-SOD1^G86R^ (Figure 5C), or ACM-TDP43^A315T^ (Figure 5D). And furthermore chronic co-incubation of spinal cord cultures with 100 nM riluzole and ACM-SOD1^G93A^ (Figure 5B) or ACM-SOD1^G86R^ (Figure 5C) completely prevented motoneuron death. The beneficial effects of this drug were less apparent on motoneurons incubated with ACM-TDP43^A315T^ (Figure 5D).

### Na_*v*_ channel blockers prevent increases in nitroxidative stress induced by ACM-SOD1^G93A^, ACM-SOD1^G86R^, or ACM-TDP43^A315T^

**Figure 6:**
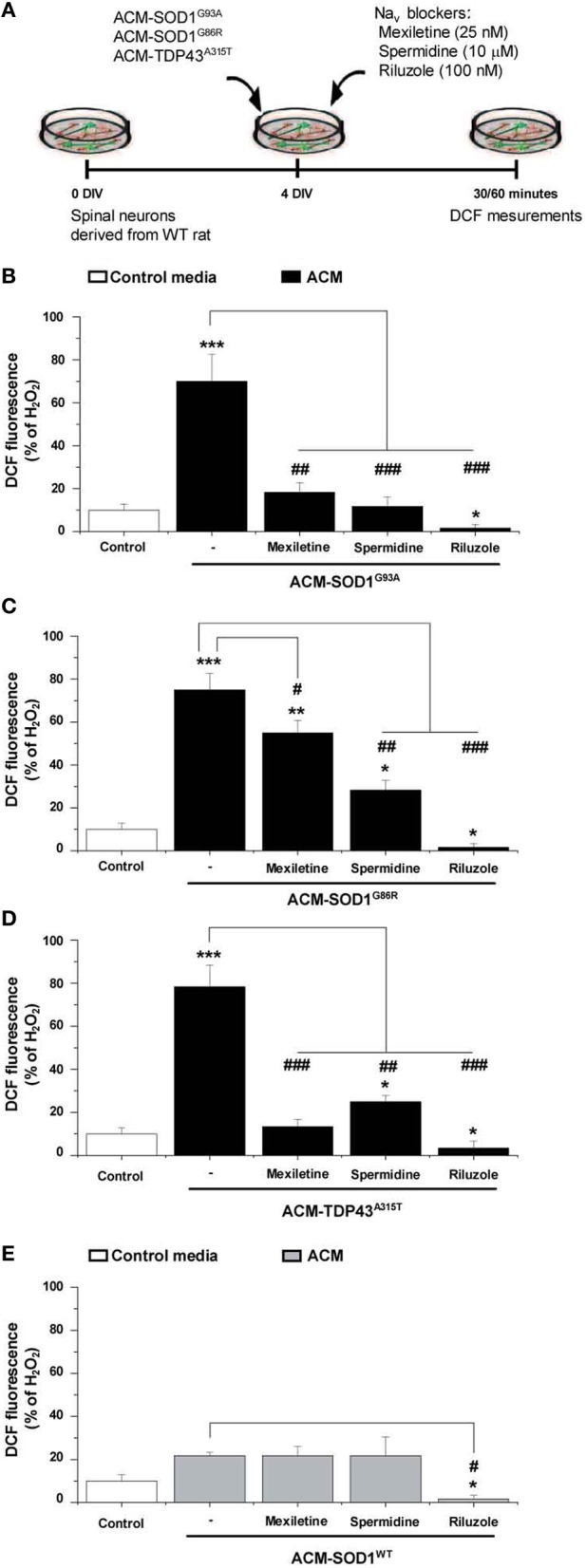
**Na_*v*_ channel blockers reduce DCF fluorescence induced by ACM-SOD1^G93A^, ACM-SOD1^G86R^, and ACM-TDP43^A315T^. (A)** Flow diagram of experiment. Spinal cultures (4 DIV) were exposed for 30 (ACM-SOD1^G93A^ or ACM-TDP43^A315T^) or 60 (ACM-SOD1^G86R^) minutes (see Figure 2 for peak of DCF fluorescence), alone or together with Na_*v*_ channel blockers mexiletine (25 nM), spermidine (10 μ M), or riluzole (100 nM). Next, cultures were incubated with the membrane permeable ROS/RNS probe CM-H_2_DCF-DA and DCF fluorescence was measured 30 min later. **(B–E)** Graphs showing the percentage of DCF fluorescent cells after being treated with the diverse Na_*v*_ channel blockers and Na_*v*_ channel blockers and ACM-SOD1^G93A^ **(B)**, ACM-SOD1^G86R^ **(C)**, ACM-SOD^WT^ **(D)**, ACM-TDP43^A315T^ **(E)**. In all experiment, H_2_O_2_ (200 μM for 20 min) served as positive control and to normalize the number of DCF-positive cells after ACM application. The graphs indicate statistics relative to motoneurons from sister cultures treated with control medium (indicated with^*^) or with only the ACM (indicated with^#^). Values represent means ± s.e.m. from at least 3 independent experiments, analyzed by One-Way ANOVA followed by a Tukey *post-hoc* test. ^*^P < 0.05, ^**^P < 0.01, ^***^P < 0.001 relative to DCF fluorescence with control media at 7 DIV; ^##^*P* < 0.01 and ^###^*P* < 0.001 compared to DCF fluorescence with ALS-causing ACM at 7 DIV.

To determine whether the Na_*v*_ channel-mediated hyperexcitability occurs upstream or downstream of the nitroxidative stress detected by DCF, we co-applied the diverse ACMs with Na_*v*_ channel blockers to 4 DIV cultures and measured the intensity of DCF fluorescence (Figure 6A). The incubation time of the Na_*v*_ channel blockers with the ACMs was chosen based on the maximum peak in the established time-course of DCF fluorescence for each conditioned media (see Figure 2): ACM-SOD1^G93A^ and ACM-TDP43^A315T^ were tested at 30 min, whereas ACM-SOD1^G86R^ was tested at 60 min. We found that all three Na_*v*_ channel blockers significantly reduced DCF fluorescence induced by the diverse ACMs, but to different degrees: mexiletine and spermidine reduced the DCF fluorescent signal induced by ACMs-SOD1^G93A^ to a level similar to that achieved in untreated cultures, while riluzole decreased DCF fluorescence to below basal levels (Figure 6B). Similar effects were observed when these Na_*v*_ channel blockers were co-applied with ACM-SOD1^G86R^ (Figure 6C), ACM-TDP43^A315T^ (Figure 6D), or ACM-SOD1^WT^ (Figure 6E). Collectively, these data indicate that astrocytes expressing diverse ALS-causing mutant genes, including in the alleles SOD1 and TDP43, secrete soluble factors that kill wild-type motoneurons through a common pathway that involves increased nitroxidative stress, mediated at least in part by Na_*v*_ channel activity.

## Discussion

We provide evidence to demonstrate that astrocytes expressing mutations in SOD1 and TDP43 genes trigger motoneuron pathology and death through non-cell-autonomous processes mediated by the release of soluble toxic factor(s). We show that conditioned media derived from astrocytes that express ALS-causing mutations in SOD1 (SOD1^G93A^ and SOD1^G86R^) and TDP43 (TDP43^A315T^) enhance ROS/RNS levels in neurons, and reduce motoneuron survival. We also document that application of anti-oxidants to spinal cord cultures prevents the increases in intracellular nitroxidative stress induced by diverse ACMs, and counteracts motoneuron death induced by the toxins in these media. Our finding that addition of Na_*v*_ channel blockers to spinal cord cultures also strongly diminish ACM-induced enhancement of DCF fluorescence and motoneuron death further indicates that Na_*v*_ channel-mediated excitability occurs upstream of nitroxidative stress and is required for the generation of a certain type of such stress. Collectively, these results indicate that astrocytes that express ALS-causing mutants in SOD1 and TDP43 contribute to ALS pathogenesis by activating a common molecular pathway that involves nitroxidative stress mediated, at least in part, by Na_*v*_ channel activity.

### Mutations in SOD1 and TDP43 cause motoneuron pathology and death by non-cell-autonomous processes

Ample evidence reveals that astrocytes expressing SOD1 mutants contribute to the pathogenesis of ALS: we and others have also demonstrated that such astrocytes release neurotoxic factors that kill primary motoneurons in culture (Nagai et al., 2007; Cassina et al., 2008; Castillo et al., 2013; Fritz et al., 2013). In agreement with these studies, we show here that ACM-SOD1^G93A^ and ACM-SOD1^G86R^ also extensively and selectively kill primary motoneurons. Additionally, we also examined whether mutants in TDP43 contribute to disease pathogenesis by non-cell-autonomous processes. As for ACM-SOD1^G93A^ and ACM-SOD1^G86R^, we document that exposure of wild-type spinal cord cultures to conditioned media generated by astrocytes derived from transgenic mice that express mutant TDP43^A315T^ suffices to trigger robust death of motoneurons. These data indicate that astrocytes harboring mutant TDP43^A315T^ release soluble toxic factor(s) into the media and thereby contribute to disease pathogenesis via non-cell-autonomous processes.

Our findings are in contrast to a recent publication by Serio et al. (2013), who showed that astrocytes expressing mutant TDP43^M337V^contribute to ALS pathology exclusively via cell-autonomous processes, and do not via non-cell-autonomous toxicity. These authors generated astrocytes from induced pluripotent stem cells (IPSCs) derived from a human ALS patient carrying the TDP43^M337V^ mutation. They report that expression of the TDP43 mutant reduced survival of the human astrocytes, but that co-culturing layers of these TDP43^M337V^ mutant containing astrocytes with wild-type motoneurons does not lead to motoneuron death. The reason(s) underlying the difference between their results and ours may be related to technical issues associated with the generation of astrocyte populations from human IPSCs, or to the particular TDP43 mutant involved. Additional experiments on the effects that diverse TDP43 mutants expressed in primary rodent astrocytes, as well as in human IPSCs cell lines, have on motoneurons are needed to resolve this discrepancy.

### Exposure of motoneurons to ACM from astrocytes carrying mutations in SOD1 and TDP43 activates a common pathogenic pathway

Several hypotheses, involving the influence of nitroxidative stress, glutamate excitotoxicity, hyperexcitability, formation of high-molecular-weight aggregates, mitochondrial dysfunction, cytoskeletal disruption, and activation of cell death signals, have been proposed to explain the toxic effect of mutated SOD1 (Beckman et al., 2001; Cleveland and Rothstein, 2001; Bruijn et al., 2004; Pasinelli and Brown, 2006; Cozzolino et al., 2012; van Zundert et al., 2012). Our results point to critical roles for nitroxidatve stress as well as Na_*v*_ channel activity in inducing motoneuron death. We and others have previously used cell cultures and slice preparations obtained from transgenic mice expressing mutations in SOD1 to report that Na_*v*_ channel activity and/or excitability is increased in motoneurons (Kuo et al., 2005; van Zundert et al., 2008; Pambo-Pambo et al., 2009; Pieri et al., 2009; Schuster et al., 2011; Quinlan et al., 2011 and reviewed in ElBasiouny et al., 2010 and van Zundert et al., 2012). Moreover, using the ACM-SOD1^G93A^ model system, we recently found that neuronal hyperexcitability, mediated at least in part through elevated Na_*v*_ channel activity, is essential for inducing motoneuron death. And furthermore, the data presented here indicate that soluble factor(s) secreted by astrocytes carrying other ALS-causing mutations in SOD1 (SOD^G86R^), and moreover in TDP43 (TDP43^A315T^), kill motoneurons via activation of Na_*v*_ channels.

Soluble mediator(s) secreted by astrocytes with mutations in SOD1 and TDP43 rapidly enhance neuronal nitroxidative stress, and lead to extensive motoneuron death within a matter of days. The rapid elevation in ROS/RNS levels observed in neurons exposed to the toxic ACMs could be due to diffusion of nitroxidative stress generated outside the cell into the motoneurons, or via intracellular *de novo* generation of ROS/RNS. We favor the second option, as the robust increase in the intensity of DCF fluorescence in motoneurons upon application of toxic ACMs is abolished when Na_*v*_ channel blockers are applied to the spinal neuron cultures. Note, however, that it has been documented that DCF detects only particular types of reactive species, including hydrogen peroxide (H_2_O_2_; in combination with cellular peroxidases), hydroxyl radicals (^•^OH), and peroxynitrite (ONOO^−^) (Estévez et al., 1999; Myhre et al., 2003; Gomes et al., 2005; Martin et al., 2007; Kalyanaraman et al., 2012), while it is seems insensitive to nitric oxide (NO) and superoxide (O^•−^_2_) (Myhre et al., 2003). It is thus possible that neurons are targets of certain ROS/RNS, including external NO or O^•−^_2_, that contribute to neuronal nitroxidative stress without being detected by the DCF probe.

With regard to this idea, amply evidence indicates that NO and O^•−^_2_ are produced by ALS glial cells—as a result of either mitochondrial dysfunction, increased NADPH oxidase activity, or inflammation—and play a pivotal role in motoneuron pathology and death (Carter et al., 2009; Drechsel et al., 2012). For example, Barbeito and collaborators (Vargas et al., 2006; Cassina et al., 2008) report that 40% of motoneurons are lost when they are co-cultured on astrocytes carrying mutated SOD1^G93A^ and that these astrocytes were found to produce excessive levels of NO and mitochondrial O^•−^_2_. Moreover, cell death is abrogated when these astrocytes are pre-treated with either anti-oxidants capable of reducing O^•−^_2_ production, or with inhibitors of NO synthase (NOS) (Vargas et al., 2006; Cassina et al., 2008). Additional studies also indicate that astrocytes and microglia that express mutated SOD1 can generate NO and NADPH oxidase (Nox)-derived ROS (Harraz et al., 2008; Marchetto et al., 2008). Also, *in vivo* production of harmful Nox-derived O^•−^_2_ is reported in human SALS patients as well as in the SOD1^G93A^ transgenic mouse model (Wu et al., 2006; Marden et al., 2007; Harraz et al., 2008). And finally, recent studies indicate that even extracellular mutant SOD1 and oxidized/misfolded SOD1 protein (both of which are likely to be secreted by cells) can activate microglia and induce nitroxidative stress (Urushitani et al., 2006; Ezzi et al., 2007; Zhao et al., 2010). Based on these studies, it is plausible that wild-type neurons are targets of external NO or O^•−^_2_ that (1) accumulates in media conditioned by astrocytes carrying ALS mutants, or (2) is generated by surrounding wild-type glial cells (astrocytes and/or microglia) within the spinal cord culture via the action of factors present in the ACM (such as mutated or oxidized/misfolded SOD1 that is secreted by the ALS astrocytes). The presence of these RNS/ROS not necessarily affects externally positioned molecules located in or on the cell membranes of neurons: Thus, NO freely diffuses across cell membranes, and whereas membranes are relatively impermeable to O^•−^_2_, recent studies indicate that this oxidative specie could permeate across redoxosomal membranes through undefined chloride channels (Mumbengegwi et al., 2008; Carter et al., 2009). On the basis of our findings presented here and in previous studies, we support the view that induction of Na_*v*_ channel activation by toxic ACMs is a central factor in initiating motoneuron death in ALS (van Zundert et al., 2012; Fritz et al., 2013). Na_*v*_ channel activity can be increased directly by different oxidative species (Hammarström and Gage, 2000; Kassmann et al., 2008; Nani et al., 2010), but also indirectly by other factors such kinases (e.g., PKC) and the Na_*v*_ channel β-subunits (Franceschetti et al., 2000; Goldin, 2003; Aman et al., 2009). It would be interesting to define by mutagenesis whether oxidation of particular amino acids residues (especially methionines and cysteines) influence Na_*v*_ channel activation; these types of experiments, however, are beyond the scope of this study.

Here we present the following hypothesis to reconcile the studies by others and the data we have present here and previously (Fritz et al., 2013) showing that ACM-SOD1^G93A^ rapidly (30 min) increases the frequency of calcium transients in cultured spinal cord neurons. Conditioned media derived from astrocytes carrying SOD1 and TDP43 mutants contains NO and/or leads to the generation of NO in spinal cord cultures. This NO in turn induces the activation of Na_*v*_ channels, leading to excessive calcium influxes through activated Ca_*v*_ channels; the calcium-mediated activation of mitochondrial respiratory chain complexes and/or Nox complexes will produce intracellular O^•−^_2_ that can interact with NO to spontaneously generate ONOO^−^ that hence can be detected by DCF and promotes intracellular damage, including protein nitration (Beckman et al., 2001; Cleveland and Rothstein, 2001; Martin et al., 2007). This hypothesis is supported by the fact that limiting the levels of O^•−^_2_ by either reducing Nox activity (Wu et al., 2006; Harraz et al., 2007) or by application of compounds such as Trolox and esculetin (in this study), which have antioxidant and free radical scavenger capacities (Barber et al., 2009), prevented motoneuron death in diverse ALS models. The finding that the antioxidant resveratrol, unlike Trolox and esculetin, was unable to significantly prevent ACM-induced motoneuron death can be explained by the fact that this compound is a poor free radical scavenger compared to the other two compounds (Barber et al., 2009).

### Do soluble toxic factor(s) released by ALS astrocytes specifically target motoneurons?

Data presented here and previously (Vargas et al., 2006; Di Giorgio et al., 2007; Nagai et al., 2007; Cassina et al., 2008; Marchetto et al., 2008; Castillo et al., 2013; Fritz et al., 2013) show compelling evidence that whereas astrocytes expressing ALS-linked mutations kill motoneurons in spinal cord cultures, they do not affect the survival of spinal cord interneurons. Data shown here and previously (Castillo et al., 2013; Fritz et al., 2013) indicate that independent of neuronal degeneration exposure of spinal cord cultures to toxic ACMs causes pathophysiological changes—including increases in nitroxidative stress, Na_*v*_ channel activity, neuronal excitability, and intracellular calcium transients—in both motoneurons and interneurons. Moreover, detailed analyses in SOD1-ALS mice also indicate that several pathological changes can be detected in interneurons, and importantly, much before the onset of disease symptoms (Martin et al., 2007; van Zundert et al., 2008, 2012; Ramírez-Jarquín et al., 2013; Wootz et al., 2013). If both types of neurons are affected in ALS, why then are motoneurons killed and interneurons spared? Using electrophysiological recordings, we recently showed (Fritz et al., 2013) that application of ACM-SOD1^G93A^ significantly increased the persistent sodium inward current (PC_Na_) of neurons; this PC_Na_ is mediated by Na_*v*_ channels (PC_Na_ is greater for Nav_1.1_ and Nav_1.6_ as compared to Nav_1.2_ and Nav_1.3_), and small increases can have important effects on neuronal excitability, leading to excessive influxes of calcium and sodium (ElBasiouny et al., 2010; van Zundert et al., 2012). Interestingly, we found that neurons with a large soma (>20 μm diameter) and expressing ≥5 primary dendrites (typical for motoneurons) displayed a larger PC_Na_ compared to neurons with a smaller soma (<20 μm diameter) and expressing ≤4 primary dendrites (typical for interneurons). Moreover, application of ACM-SOD1^G93A^ further increased the PC_Na_ of neurons, especially of motoneurons. The expression of specific Na_*v*_ channel subtypes, and subsequent activation of these channels by soluble toxic factor(s) released by ALS astrocytes, will thus induce larger PC_Na_ and in turn cause more sustained calcium influxes in motoneurons compared to interneurons. In addition, because motoneurons have a limited cytosolic calcium-buffering capacity, excessive uptake of calcium by mitochondria would be an initial step in a cascade of events that impair mitochondrial function, thereby likely producing excessive O^•−^_2_ that ultimately leads to motoneuron death (von Lewinski and Keller, 2005; Bento-Abreu et al., 2010; van Zundert et al., 2012).

In summary, we have elucidated critical roles for both nitroxidative stress and Na_*v*_ channel activation in the death of motoneuron that is induced in diverse ALS models. Finally, because patients with SALS and FALS display a similar pathology, have comparable clinical symptoms that include hyperexcitability, and experience a beneficial effect of riluzole, it is possible that diverse Na_*v*_ channel blockers will show benefit in both forms of ALS.

## Author contributions

Fabiola Rojas, Nicole Cortes, and Sebastian Abarzua performed MN survival experiments. Nicole Cortes and Fabiola Rojas performed DCF measurement. All (Fabiola Rojas, Nicole Cortes, Sebastian Abarzua, Agnieszka Dyrda, and Brigitte van Zundert) analyzed the data and wrote the manuscript.

### Conflict of interest statement

The authors declare that the research was conducted in the absence of any commercial or financial relationships that could be construed as a potential conflict of interest.

## Supplementary material

Supplementary Figure 1 **Exposure of primary spinal cord cultures to Control media or ACM-NT-Control media does not induce increases in intracellular DCF fluorescence. (A)** Flow diagram of experiment. Primary wild-type spinal cultures (4 DIV) were exposed for 0–120 min with the different medias (solid lines), washed to remove the ACMs (filled circles), and loaded for 30 min with the fluorescent membrane permeable ROS/RNS probe CM-H_2_DCF-DA (dotted lines). Next, cultures were washed and DCF measurements were immediately performed (filled triangles). **(B–D)** Cultures were exposed to three types of control media: “Control” media that was not conditioned by astrocytes **(B)**, “ACM-SOD1^WT^” media derived from astrocytes that were harvested from transgenic mice carrying the non-pathological human wild-type SOD1 gene **(C)**, and “ACM-NT-Control” media derived from astrocytes that were harvested from littermate mice that were negative for the mutant SOD1 or TDP43 gene; here we show media from TDP43^A315T−/−^ astrocytes. Similar results were obtained after application of media from SOD1^G93A−/−^ or SOD1^G86R−/−^ astrocytes **(D)**. The negatives of representative DCF fluorescent images (in which both motoneurons and interneurons are marked) and corresponding phase contrast images of spinal cord cultures photographed at the different indicated time points of media application are shown. Note that unlike H_2_O_2_ (200 μ M for 20 min), which served as a positive control (shown in **B** in the right), none of the control media induced increases in intracellular DCF fluorescence. Scale bar, 200 μm.

Supplementary Figure 2 **Time-course of motoneuron survival in primary spinal cord cultures exposed to ACM-SOD1**^G93A^. Primary wild-type (WT) rat spinal cord cultures (4 DIV) were exposed for 2, 8, 24, and 72 h to ACM derived from transgenic mice overexpressing SOD1^G93A^ (ACM-SOD1^G93A^) and fixed immediately afterwards. Cell survival was assayed with immunocytochemistry. Graph showing the percentage of motoneurons that survived after treatment with ACMSOD1^G93A^, relative to control medium and with media derived from transgenic mice carrying the non-pathological human wild-type SOD1 gene (ACM-SOD1^WT^). Survival is shown relative to cultures treated with control media. Values represent mean ± s.e.m. from at least 3 independent experiments performed in duplicate, analyzed by One-Way ANOVA followed by a Tukey *post-hoc* test. ^***^*P* < 0.001 relative to control medium at 7 DIV.

Supplementary Figure 3 **Time-course of DCF fluorescence in spinal cord cultures exposed to ACM-SOD1**^G93A^, **ACM-SOD1**^G86R^, **or ACM-TDP43**^A315T^. **(A)** Flow diagram of experiment. Primary wild-type spinal cultures (4 DIV) were exposed for 0, 30, 60, 90, and 120 min and 6, 24, 48, and 72 h with the different ACMs (solid lines), washed to remove the ACMs (filled circles), and loaded for 30 min with the fluorescent membrane permeable ROS/RNS probe CM-H_2_DCF-DA (dotted lines). Next, cultures were washed and DCF measurements were immediately performed (filled triangles). **(B–D)** Graphs showing the percentage of DCF fluorescent cells (including both motoneurons and interneurons) in cultures exposed to ACM-SOD1^G93A^ **(B)**, ACM-SOD1^G86R^ **(C)**, or ACM-TDP43^A315T^ **(D)**. Results obtained with different controls are included in the graphs: ACMSOD1^WT^ **(B)**, ACM-NT-Control from SOD1^G86R−/−^astrocytes **(C),** and ACM-NT-Control from TDP43^A314T−/−^ astrocytes **(D)**. DCF fluorescence is relative to cultures treated with H_2_O_2_, 200 μ M for 20 min (100%). Note that application of toxic ACMs resulted in an initial peak of DCF fluorescence at 30–60 min that was followed by a decline in the DCF signal which then started to steadily increase from 24 h on. Values represent mean ± s.e.m. from at least 3 independent experiments performed in duplicate, analyzed by *t*-test. ^*^*p* < 0.05, ^**^*p* < 0.01, ^***^*p* < 0.001 vs. control.

Supplementary Figure 4 **Anti-oxidant resveratrol is as effective as Trolox and esculetin in reducing DCF fluorescence induced by H_2_O_2_. (A)** Flow diagram of experiment. Primary spinal cord cultures (4 DIV) were exposed to 200 μ M H_2_O_2_ for 20 min in the presence or absence of anti-oxidants. Cells were washed and then loaded with the membrane permeable ROS/RNS probe CM-H_2_DCF-DA for 30 min. After washing, DCF fluorescence was measured immediately. **(B)** Graphs showing the percentage of DCF fluorescent cells (including motoneurons and interneurons) in cultures exposed to H_2_O_2_ and Trolox (1 μ M), esculetin (25 μ M), or resveratrol (1 μ M). Values represent means ± s.e.m. from at least 3 independent experiments, analyzed by One-Way ANOVA followed by a Tukey *post-hoc* test. ^##^*P* < 0.01, ^###^*P* < 0.001 relative to DCF fluorescence in H_2_O_2_ treated cultures.

Supplementary Table 1 **Effects of a wide range of concentrations of antioxidants on the survival of control motoneurons and those exposed to ACM-SOD1**^*G*93*A*^. **(A)** Analysis of survival of motoneurons at 7 DIV in primary cultures after incubation for 4 days with multiple doses of the anti-oxidants Trolox, esculetin, and resveratrol. **(B)** Analysis of surviving motoneurons at 7 DIV in primary cultures after incubation for 4 days with ACM-hSOD1^G93A^ alone (no drug), or with ACM plus different doses of the anti-oxidants. In bold are indicated concentrations at which concentration the anti-oxidant was selected for further analysis on preventing the toxicity induced by the diverse ACMs; those results are displayed in Figure 3. Values represent mean ± s.e.m. from at least 3 independent experiments performed in duplicate, analyzed by *t*-test vs. control.

## References

[B1] Abel O. Powell J. F. Andersen P. M. Al-Chalabi A. (2012). ALSoD: a user-friendly online bioinformatics tool for amyotrophic lateral sclerosis genetics. Hum. Mutat. 33, 1345–1351 10.1002/humu.22157 22753137

[B2] Aman T. K. Grieco-Calub T. M. Chen C. Rusconi R. Slat E. A. Isom L. L. (2009). Regulation of persistent Na current by interactions between beta subunits of voltage-gated Na channels. J. Neurosci. 29, 2027–2042 10.1523/JNEUROSCI.4531-08.2009 19228957 PMC2667244

[B3] Appaix F. Girod S. Boisseau S. Romer J. Vial J. C. Albrieux M. (2012). Specific *in vivo* staining of astrocytes in the whole brain after intravenous injection of sulforhodamine dyes. PLoS ONE 7:e35169 10.1371/journal.pone.0035169 22509398 PMC3324425

[B4] Ascherio A. Weisskopf M. G. O'reilly E. J. Jacobs E. J. McCullough M. L. Calle E. E. (2005). Vitamin E intake and risk of amyotrophic lateral sclerosis. Ann. Neurol. 57, 104–110 10.1002/ana.20316 15529299

[B5] Barber S. C. Higginbottom A. Mead R. J. Barber S. Shaw P. J. (2009). An *in vitro* screening cascade to identify neuroprotective antioxidants in ALS. Free Radic. Biol. Med. 46, 1127–1138 10.1016/j.freeradbiomed.2009.01.019 19439221 PMC2742740

[B6] Barber S. C. Shaw P. J. (2010). Oxidative stress in ALS: key role in motor neuron injury and therapeutic target. Free Radic. Biol. Med. 48, 629–641 10.1016/j.freeradbiomed.2009.11.018 19969067

[B7] Baur J. A. Sinclair D. A. (2006). Therapeutic potential of resveratrol: the *in vivo* evidence. Nat. Rev. Drug Discov. 5, 493–506 10.1038/nrd2060 16732220

[B8] Beckman J. S. Estévez A. G. Crow J. P. Barbeito L. (2001). Superoxide dismutase and the death of motoneurons in ALS. Trends Neurosci. 24, S15–S20 10.1016/S0166-2236(00)01981-0 11881740

[B9] Bellingham M. C. (2011). A review of the neural mechanisms of action and clinical efficiency of riluzole in treating amyotrophic lateral sclerosis: what have we learned in the last decade? CNS Neurosci. Ther. 17, 4–31 10.1111/j.1755-5949.2009.00116.x 20236142 PMC6493865

[B10] Bento-Abreu A. van Damme P. van Den Bosch L. Robberecht W. (2010). The neurobiology of amyotrophic lateral sclerosis. Eur. J. Neurosci. 31, 2247–2265 10.1111/j.1460-9568.2010.07260.x 20529130

[B11] Boillée S. Yamanaka K. Lobsiger C. S. Copeland N. G. Jenkins N. A. Kassiotis G. (2006). Onset and progression in inherited ALS determined by motor neurons and microglia. Science 312, 1389–1392 10.1126/science.1123511 16741123

[B12] Bruijn L. I. Miller T. M. Cleveland D. W. (2004). Unraveling the mechanisms involved in motor neuron degeneration in ALS. Annu. Rev. Neurosci. 27, 723–749 10.1146/annurev.neuro.27.070203.144244 15217349

[B13] Carter B. J. Anklesaria P. Choi S. Engelhardt J. F. (2009). Redox modifier genes and pathways in amyotrophic lateral sclerosis. Antioxid. Redox Signal. 11, 1569–1586 10.1089/ARS.2008.2414 19187001 PMC2842588

[B14] Cassina P. Cassina A. Pehar M. Castellanos R. Gandelman M. de León A. (2008). Mitochondrial dysfunction in SOD1G93A-bearing astrocytes promotes motor neuron degeneration: prevention by mitochondrial-targeted antioxidants. J. Neurosci. 28, 4115–4122 10.1523/JNEUROSCI.5308-07.2008 18417691 PMC3844766

[B15] Castillo K. Nassif M. Valenzuela V. Rojas F. Matus S. Mercado G. (2013). Trehalose delays the progression of amyotrophic lateral sclerosis by enhancing autophagy in motoneurons. Autophagy 9, 1308–1320 10.4161/auto.25188 23851366

[B16] Catterall W. A. Goldin A. L. Waxman S. G. (2005). International Union of Pharmacology. XLVII. Nomenclature and structure-function relationships of voltage-gated sodium channels. Pharmacol. Rev. 57, 397–409 10.1124/pr.57.4.4 16382098

[B17] Clement A. M. Nguyen M. D. Roberts E. A. Garcia M. L. Boillée S. Rule M. (2003). Wild-type nonneuronal cells extend survival of SOD1 mutant motor neurons in ALS mice. Science 302, 113–117 10.1126/science.1086071 14526083

[B18] Cleveland D. W. Rothstein J. D. (2001). From Charcot to Lou Gehrig: deciphering selective motor neuron death in ALS. Nat. Rev. Neurosci. 2, 806–819 10.1038/35097565 11715057

[B19] Cozzolino M. Pesaresi M. G. Gerbino V. Grosskreutz J. Carrì M. T. (2012). Amyotrophic lateral sclerosis: new insights into underlying molecular mechanisms and opportunities for therapeutic intervention. Antioxid. Redox Signal. 17, 1277–1330 10.1089/ars.2011.4328 22413952

[B20] Desnuelle C. Dib M. Garrel C. Favier A. (2001). A double-blind, placebo-controlled randomized clinical trial of alpha-tocopherol (vitamin E) in the treatment of amyotrophic lateral sclerosis. ALS riluzole-tocopherol Study Group. Amyotroph. Lateral Scler. Other Motor Neuron Disord. 2, 9–18 10.1080/146608201300079364 11465936

[B30] Di Giorgio F. P. Carrasco M. A. Siao M. C. Maniatis T. Eggan K. (2007). Non-cell autonomous effect of glia on motor neurons in an embryonic stem cell-based ALS model. Nat. Neurosci. 10, 608–614 10.1038/nn1885 17435754 PMC3139463

[B21] Distelmaier F. Valsecchi F. Forkink M. van Emst-de Vries S. Swarts H. G. Rodenburg R. J. (2012). Trolox-sensitive reactive oxygen species regulate mitochondrial morphology, oxidative phosphorylation and cytosolic calcium handling in healthy cells. Antioxid. Redox Signal. 17, 1657–1669 10.1089/ars.2011.4294 22559215 PMC3474189

[B22] Drechsel D. A. Estévez A. G. Barbeito L. Beckman J. S. (2012). Nitric oxide-mediated oxidative damage and the progressive demise of motor neurons in ALS. Neurotox. Res. 22, 251–264 10.1007/s12640-012-9322 22488161 PMC4145402

[B23] ElBasiouny S. M. Schuster J. E. Heckman C. J. (2010). Persistent inward currents in spinal motoneurons: important for normal function but potentially harmful after spinal cord injury and in amyotrophic lateral sclerosis. Clin. Neurophysiol. 121, 1669–1679 10.1016/j.clinph.2009.12.041 20462789 PMC3000632

[B23a] Estévez A. G. Crow J. P. Sampson J. B. Reiter C. Zhuang Y. Richardson G. J. (1999). Induction of nitric oxide - dependent apoptosis in motor neurons by zinc-deficient superoxide dismutase. Science 286, 2498–2500 10617463 10.1126/science.286.5449.2498

[B24] Ezzi S. A. Urushitani M. Julien J. P. (2007). Wild-type superoxide dismutase acquires binding and toxic properties of ALS-linked mutant forms through oxidation. J. Neurochem. 102, 170–178 10.1111/j.1471-4159.2007.04531.x 17394546

[B25] Ferraiuolo L. Kirby J. Grierson A. J. Sendtner M. Shaw P. J. (2011). Molecular pathways of motor neuron injury in amyotrophic lateral sclerosis. Nat. Rev. Neurol. 7, 616–630 10.1038/nrneurol.2011.152 22051914

[B26] Fleidervish I. A. Goldberg Y. Ovsyshcher I. E. (2008). Bolus injection of acetylcholine terminates atrial fibrillation in rats. Eur. J. Pharmacol. 579, 326–329 10.1016/j.ejphar.2007.11.010 18078927

[B27] Franceschetti S. Taverna S. Sancini G. Panzica F. Lombardi R. Avanzini G. (2000). Protein kinase C-dependent modulation of Na^+^currents increases the excitability of rat neocortical pyramidal neurons. J. Physio. 528, 291–304 10.1111/j.1469-7793.2000.00291.x 11034619 PMC2270127

[B28] Fritz E. Izaurieta P. Weiss A. Mir F. R. Rojas P. Gonzalez D. (2013). Mutant SOD1-expressing astrocytes release toxic factors that trigger motoneuron death by inducing hyperexcitability. J. Neurophysiol. 109, 2803–2814 10.1152/jn.00500.2012 23486205 PMC3680799

[B29] Ghiselli A. Serafini M. Maiani G. Azzini E. Ferro-Luzzi A. (1995). A fluorescence-based method for measuring total plasma antioxidant capability. Free Radic. Biol. Med. 18, 29–36 10.1016/0891-5849(94)00102-P 7896168

[B31] Goldin A. L. (2003). Mechanisms of sodium channel inactivation. Curr. Opin. Neurobiol. 13, 284–290 10.1016/S0959-4388(03)00065-5 12850212

[B32] Gomes A. Fernandes E. Lima J. L. (2005). Fluorescence probes used for detection of reactive oxygen species. J. Biochem. Biophys. Methods 65, 45–80 10.1016/j.jbbm.2005.10.003 16297980

[B33] Graf M. Ecker D. Horowski R. Kramer B. Riederer P. Gerlach M. (2005). High dose vitamin E therapy in amyotrophic lateral sclerosis as add-on therapy to riluzole: results of a placebo-controlled double-blind study. J. Neural Transm. 112, 649–660 10.1007/s00702-004-0220-1 15517433

[B34] Gurney M. E. Cutting F. B. Zhai P. Doble A. Taylor C. P. Andrus P. K. (1996). Benefit of vitamin E, riluzole, and gabapentin in a transgenic model of familial amyotrophic lateral sclerosis. Ann. Neurol. 39, 147–157 10.1002/ana.410390203 8967745

[B35] Gurney M. E. Pu H. Chiu A. Y. Dal Canto M. C. Polchow C. Y. Alexander D. D. (1994). Motor neuron degeneration in mice that express a human Cu, Zn superoxide dismutase mutation. Science 264, 1772–1775 10.1126/science.8209258 8209258

[B36] Haidet-Phillips A. M. Hester M. E. Miranda C. J. Meyer K. Braun L. Frakes A. (2011). Astrocytes from familial and sporadic ALS patients are toxic to motor neurons. Nat. Biotechnol. 29, 824–828 10.1038/nbt.1957 21832997 PMC3170425

[B37] Hammarström A. K. Gage P. W. (2000). Oxygen-sensing persistent sodium channels in rat hippocampus. J. Physiol. 529, 107–118 10.1111/j.1469-7793.2000.00107.x 11080255 10.1111/j.1469-7793.2000.00107.x PMC2270177

[B38] Harraz M. M. Marden J. J. Zhou W. Zhang Y. Williams A. Sharov V. S. (2008). SOD1 mutations disrupt redox-sensitive Rac regulation of NADPH oxidase in a familial ALS model. J. Clin. Invest. 118, 659–670 10.1172/JCI34060 18219391 PMC2213375

[B38a] Harraz M. M. Park A. Abbott D. Zhou W. Zhang Y. Engelhardt J. F. (2007). MKK6 phosphorylation regulates production of superoxide by enhancing Rac GTPase activity. Antioxid. Redox Signal. 9, 1803–1813 10.1089/ars.2007.1579 17854274 PMC3597076

[B39] Ilieva H. Polymenidou M. Cleveland D. W. (2009). Non-cell autonomous toxicity in neurodegenerative disorders: ALS and beyond. J. Cell Biol. 187, 761–772 10.1083/jcb.200908164 19951898 PMC2806318

[B40] Kalyanaraman B. Darley-Usmar V. Davies K. J. Dennery P. A. Forman H. J. Grisham M. B. (2012). Measuring reactive oxygen and nitrogen species with fluorescent probes: challenges and limitations. Free Radic. Biol. Med. 52, 1–6 10.1016/j.freeradbiomed.2011.09.030 22027063 PMC3911769

[B41] Kaneko T. Tahara S. Takabayashi F. (2003). Suppression of lipid hydroperoxide-induced oxidative damage to cellular DNA by esculetin. Biol. Pharm. Bull. 26, 840–844 10.1248/bpb.26.840 12808296

[B42] Kassmann M. Hansel A. Leipold E. Birkenbeil J. Lu S. Q. Hoshi T. (2008). Oxidation of multiple methionine residues impairs rapid sodium channel inactivation. Pflugers Arch. 456, 1085–1095 10.1007/s00424-008-0477-6 18369661 PMC2913308

[B43] Khaldy H. Escames G. León J. Vives F. Luna J. D. Acuña-Castroviejo D. (2000). Comparative effects of melatonin, L-deprenyl, Trolox and ascorbate in the suppression of hydroxyl radical formation during dopamine autoxidation *in vitro*. J. Pineal Res. 29, 100–107 10.1034/j.1600-079X.2000.290206.x 10981823

[B45] Kuo J. J. Siddique T. Fu R. Heckman C. J. (2005). Increased persistent Na+ current and its effect on excitability in motoneurones cultured from mutant SOD1 mice. J. Physiol. 563, 843–854 10.1113/jphysiol.2004.074138 15649979 PMC1665614

[B46] Igaz L. M. Kwong L. K. Lee E. B. Chen-Plotkin A. Swanson E. Unger T. (2011). Dysregulation of the ALS-associated gene TDP-43 leads to neuronal death and degeneration in mice. J. Clin. Invest. 121, 726–738 10.1172/JCI44867 21206091 PMC3026736

[B47] Lin W. L. Wang C. J. Tsai Y. Y. Liu C. L. Hwang J. M. Tseng T. H. (2000). Inhibitory effect of esculetin on oxidative damage induced by t-butyl hydroperoxide in rat liver. Arch. Toxicol. 74, 467–472 10.1007/s002040000148 11097384

[B61] Ling S. C. Polymenidou M. Cleveland D. W. (2013). Converging mechanisms in ALS and FTD: disrupted RNA and protein homeostasis. Neuron 79, 416–438 10.1016/j.neuron.2013.07.033 23931993 PMC4411085

[B48] Lobsiger C. S. Boillee S. McAlonis-Downes M. Khan A. M. Feltri M. L. Yamanaka K. (2009). Schwann cells expressing dismutase active mutant SOD1 unexpectedly slow disease progression in ALS mice. Proc. Natl. Acad. Sci. U.S.A. 106, 4465–4470 10.1073/pnas.0813339106 19251638 PMC2657393

[B49] Marchetto M. C. Muotri A. R. Mu Y. Smith A. M. Cezar G. G. Gage F. H. (2008). Non-cell-autonomous effect of human SOD1G37R astrocytes on motor neurons derived from human embryonic stem cells. Cell Stem Cell 3, 649–657 10.1016/j.stem.2008.10.001 19041781

[B50] Marden J. J. Harraz M. M. Williams A. J. Nelson K. Luo M. Paulson H. (2007). Redox modifier genes in amyotrophic lateral sclerosis in mice. J. Clin. Invest. 117, 2913–2319 10.1172/JCI31265 17853944 PMC1974865

[B51] Martin L. J. Liu Z. Chen K. Price A. C. Pan Y. Swaby J. A. (2007). Motor neuron degeneration in amyotrophic lateral sclerosis mutant superoxide dismutase-1 transgenic mice: mechanisms of mitochondriopathy and cell death. J. Comp. Neurol. 500, 20–46 10.1002/cne.21160 17099894

[B51a] Meyer K. Ferraiuolo L. Miranda C. J. Likhite S. McElroy S. Renusch S. (2014). Direct conversion of patient fibroblasts demonstrates non-cell autonomous toxicity of astrocytes to motor neurons in familial and sporadic ALS. Proc. Natl. Acad. Sci. U.S.A. 111, 829–832 10.1073/pnas.1314085111 24379375 PMC3896192

[B52] Mumbengegwi D. R. Li Q. Li C. Bear C. E. Engelhardt J. F. (2008). Evidence for a superoxide permeability pathway in endosomal membranes. Mol. Cell. Biol. 28, 3700–3712 10.1128/MCB.02038-07 18378695 PMC2423302

[B53] Myhre O. Andersen J. M. Aarnes H. Fonnum F. (2003). Evaluation of the probes 2',7'-dichlorofluorescin diacetate, luminol, and lucigenin as indicators of reactive species formation. Biochem. Pharmacol. 65, 1575–1582 10.1016/S0006-2952(03)00083-2 12754093

[B54] Nagai M. Re D. B. Nagata T. Chalazonitis A. Jessell T. M. Wichterle H. (2007). Astrocytes expressing ALS-linked mutated SOD1 release factors selectively toxic to motor neurons. Nat. Neurosci. 10, 615–622 10.1038/nn1876 17435755 PMC3799799

[B55] Nani F. Cifra A. Nistri A. (2010). Transient oxidative stress evokes early changes in the functional properties of neonatal rat hypoglossal motoneurons *in vitro*. Eur. J. Neurosci. 31, 951–966 10.1111/j.1460-9568.2010.07108.x 20214680

[B56] Olschewski A. Schnoebel-Ehehalt R. Li Y. Tang B. Bräu M. E. Wolff M. (2009). Mexiletine and lidocaine suppress the excitability of dorsal horn neurons. Anesth. Analg. 109, 258–264 10.1213/ane.0b013e3181a3d5d8 19535719

[B57] Pambo-Pambo A. Durand J. Gueritaud J. P. (2009). Early excitability changes in lumbar motoneurons of transgenic SOD1G85R and SOD1G(93A-Low) mice. J. Neurophysiol. 102, 3627–3642 10.1152/jn.00482.2009 19828728

[B58] Pasinelli P. Brown R. H. (2006). Molecular biology of amyotrophic lateral sclerosis: insights from genetics. Nat. Rev. Neurosci. 7, 710–723 10.1038/nrn1971 16924260

[B59] Pehar M. Cassina P. Vargas M. R. Castellanos R. Viera L. Beckman J. S. (2004). Astrocytic production of nerve growth factor in motor neuron apoptosis: implications for amyotrophic lateral sclerosis. J. Neurochem. 89, 464–473 10.1111/j.1471-4159.2004.02357.x 15056289

[B60] Pieri M. Carunchio I. Curcio L. Mercuri N. B. Zona C. (2009). Increased persistent sodium current determines cortical hyperexcitability in a genetic model of amyotrophic lateral sclerosis. Exp Neurol. 215, 368–379 10.1016/j.expneurol.2008.11.002 19071115

[B62] Quinlan K. A. Schuster J. E. Fu R. Siddique T. Heckman C. J. (2011). Altered postnatal maturation of electrical properties in spinal motoneurons in a mouse model of amyotrophic lateral sclerosis. J. Physiol. 589, 2245–2260 10.1113/jphysiol.2010.200659 21486770 PMC3098701

[B63] Ragsdale D. S. McPhee J. C. Scheuer T. Catterall W. A. (1994). Molecular determinants of state-dependent block of Na+ channels by local anesthetics. Science 265, 1724–1728 10.1126/science.8085162 8085162

[B64] Ramírez-Jarquín U. N. Lazo-Gómez R. Tovar-Y-Romo L. B. Tapia R. (2013). Spinal inhibitory circuits and their role in motor neuron degeneration. Neuropharmacology. [Epub ahead of print]. 10.1016/j.neuropharm.2013.10.003 24157492

[B65] Ripps M. E. Huntley G. W. Hof P. R. Morrison J. H. Gordon J. W. (1995). Transgenic mice expressing an altered murine superoxide dismutase gene provide an animal model of amyotrophic lateral sclerosis. Proc. Natl. Acad. Sci. U.S.A. 92, 689–693 10.1073/pnas.92.3.689 7846037 PMC42685

[B66] Schuster J. E. Fu R. Siddique T. Heckman C. J. (2011). Effect of prolonged riluzole exposure on cultured motoneurons in a mouse model of ALS. J. Neurophysiol. 107, 484–492 10.1152/jn.00714.2011 22013234 PMC3349692

[B67] Sepulveda F. J. Bustos F. J. Inostroza E. Zúñiga F. A. Neve R. L. Montecino M. (2010). Differential roles of NMDA receptor subtypes NR2A and NR2B in dendritic branch development and requirement of RasGRF1. J Neurophysiol. 103, 1758–1770 10.1152/jn.00823.2009 20107120

[B68] Serio A. Bilican B. Barmada S. J. Ando D. M. Zhao C. Siller R. (2013). Astrocyte pathology and the absence of non-cell autonomy in an induced pluripotent stem cell model of TDP-43 proteinopathy. Proc. Natl. Acad. Sci. U.S.A. 110, 4697–4702 10.1073/pnas.1300398110 23401527 PMC3607024

[B69] Theiss R. D. Kuo J. J. Heckman C. J. (2007). Persistent inward currents in rat ventral horn neurones. J. Physiol. 580, 507–522 10.1113/jphysiol.2006.124123 17289788 PMC2075552

[B70] Tucker J. M. Townsend D. M. (2005). Alpha-tocopherol: roles in prevention and therapy of human disease. Biomed. Pharmacother. 59, 380–387 10.1016/j.biopha.2005.06.005 16081238 PMC6361124

[B71] Urushitani M. Sik A. Sakurai T. Nukina N. Takahashi R. Julien J. P. (2006). Chromogranin-mediated secretion of mutant superoxide dismutase proteins linked to amyotrophic lateral sclerosis. Nat. Neurosci. 9, 108–118 10.1038/nn1603 16369483

[B72] van Zundert B. Izaurieta P. Fritz E. Alvarez F. J. (2012). Early pathogenesis in the adult-onset neurodegenerative disease amyotrophic lateral sclerosis. J. Cell Biochem. 113, 3301–3312 10.1002/jcb.24234 22740507 PMC3886915

[B73] van Zundert B. Peuscher M. H. Hynynen M. Chen A. Neve R. L. Brown R. H. Jr. (2008). Neonatal neuronal circuitry shows hyperexcitable disturbance in a mouse model of the adult-onset neurodegenerative disease amyotrophic lateral sclerosis. J. Neurosci. 28, 10864–10874 10.1523/JNEUROSCI.1340-08.2008 18945894 PMC3844745

[B74] Vargas M. R. Pehar M. Cassina P. Beckman J. S. Barbeito L. (2006). Increased glutathione biosynthesis by Nrf2 activation in astrocytes prevents p75NTR-dependent motor neuron apoptosis. J. Neurochem. 97, 687–696 10.1111/j.1471-4159.2006.03742.x 16524372

[B75] von Lewinski F. Keller B. U. (2005). Ca2+, mitochondria and selective motoneuron vulnerability: implications for ALS. Trends Neurosci. 28, 494–500 10.1016/j.tins.2005.07.001 16026864

[B76] Wegorzewska I. Bell S. Cairns N. J. Miller T. M. Baloh R. H. (2009). TDP-43 mutant transgenic mice develop features of ALS and frontotemporal lobar degeneration. Proc. Natl. Acad. Sci. U.S.A. 106, 18809–18814 10.1073/pnas.0908767106 19833869 PMC2762420

[B44] Wils H. Kleinberger G. Janssens J. Pereson S. Joris G. Cuijt I. (2010). TDP-43 transgenic mice develop spastic paralysis and neuronal inclusions characteristic of ALS and frontotemporal lobar degeneration. Proc. Natl. Acad. Sci. U.S.A. 107, 3858–3863 10.1073/pnas.0912417107 20133711 PMC2840518

[B44a] Williams K. (1997). Modulation and block of ion channels: a new biology of polyamines. Cell Signal. 9, 1–13 10.1016/S0898-6568(96)00089-7 9067625

[B77] Wootz H. Fitzsimons-Kantamneni E. Larhammar M. Rotterman T. M. Enjin A. Patra K. (2013). Alterations in the motor neuron-renshaw cell circuit in the Sod1(G93A) mouse model. J. Comp. Neurol. 521, 1449–1469 10.1002/cne.23322 PMC3604165 23172249

[B78] Wu D. C. Re D. B. Nagai M. Ischiropoulos H. Przedborski S. (2006). The inflammatory NADPH oxidase enzyme modulates motor neuron degeneration in amyotrophic lateral sclerosis mice. Proc. Natl. Acad. Sci. U.S.A. 103, 12132–12137 10.1073/pnas.0603670103 16877542 PMC1562547

[B79] Yamanaka K. Chun S. J. Boillee S. Fujimori-Tonou N. Yamashita H. Gutmann D. H. (2008a). Astrocytes as determinants of disease progression in inherited amyotrophic lateral sclerosis. Nat. Neurosci. 11, 251–253 10.1038/nn2047 18246065 PMC3137510

[B80] Yamanaka K. Boillee S. Roberts E. A. Garcia M. L. McAlonis-Downes M. Mikse O. R. (2008b). Mutant SOD1 in cell types other than motor neurons and oligodendrocytes accelerates onset of disease in ALS mice. Proc. Natl. Acad. Sci. U.S.A. 105, 7594–7599 10.1073/pnas.0802556105 18492803 PMC2396671

[B81] Zhao W. Beers D. R. Henkel J. S. Zhang W. Urushitani M. Julien J. P. (2010). Extracellular mutant SOD1 induces microglial-mediated motoneuron injury. Glia 58, 231–243 10.1002/glia.20919 19672969 PMC2784168

