# Supplementary figures and images for: Astrocytes expressing mutant SOD1 and TDP43 trigger motoneuron death that is mediated via sodium channels and nitroxidative stress
Source: Front Cell Neurosci. 2014 Feb 7;8:24. doi: 10.3389/fncel.2014.00024 (PMC3916762; doi:10.3389/fncel.2014.00024)

**A**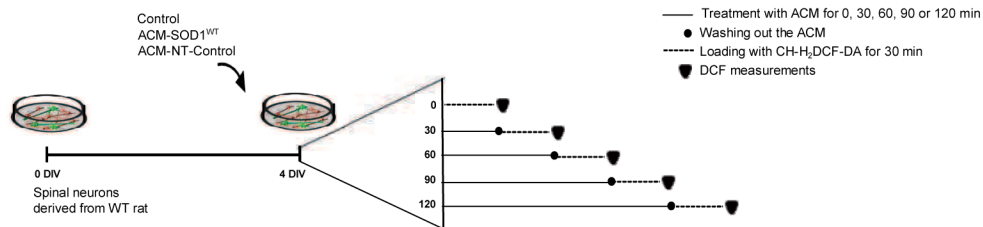**B**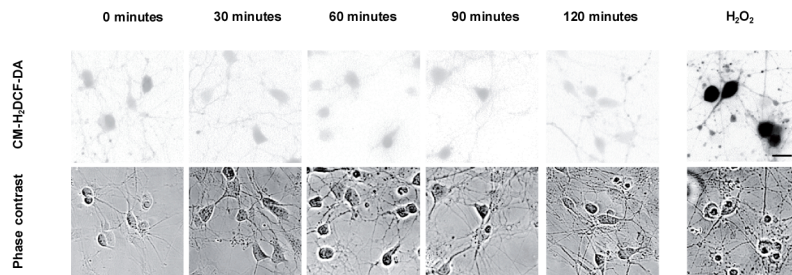**C**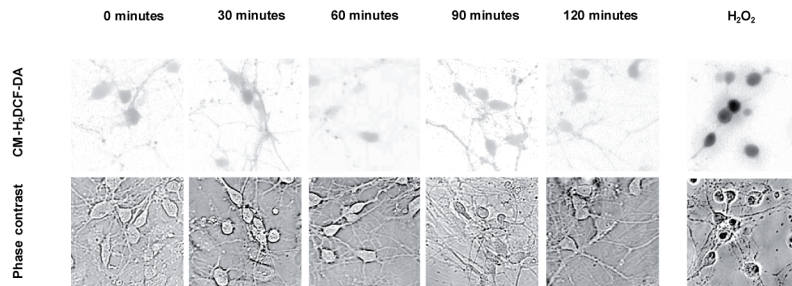**D**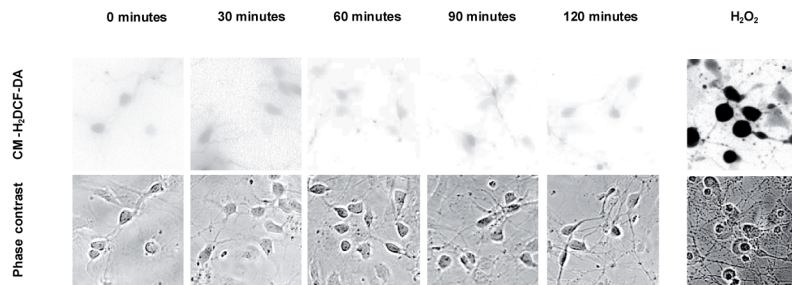

Supplement: Supplementary Figure 1 — Exposure of primary spinal cord cultures to Control media or ACM-NT-Control media does not induce increases in intracellular DCF fluorescence. (A) Flow diagram of experiment. Primary wild-type spinal cultures (4 DIV) were exposed for 0–120 min with the different medias (solid lines), washed to remove the ACMs (filled circles), and loaded for 30 min with the fluorescent membrane permeable ROS/RNS probe CM-H2DCF-DA (dotted lines). Next, cultures were washed and DCF measurements were immediately performed (filled triangles). (B–D) Cultures were exposed to three types of control media: “Control” media that was not conditioned by astrocytes (B), “ACM-SOD1WT” media derived from astrocytes that were harvested from transgenic mice carrying the non-pathological human wild-type SOD1 gene (C), and “ACM-NT-Control” media derived from astrocytes that were harvested from littermate mice that were negative for the mutant SOD1 or TDP43 gene; here we show media from TDP43A315T−/− astrocytes. Similar results were obtained after application of media from SOD1G93A−/− or SOD1G86R−/− astrocytes (D). The negatives of representative DCF fluorescent images (in which both motoneurons and interneurons are marked) and corresponding phase contrast images of spinal cord cultures photographed at the different indicated time points of media application are shown. Note that unlike H2O2 (200 μ M for 20 min), which served as a positive control (shown in B in the right), none of the control media induced increases in intracellular DCF fluorescence. Scale bar, 200 μm. [file Presentation1.PDF]

Control media

ACM-SOD1<sup>G93A</sup>

ACM-SOD1<sup>WT</sup>

Motoneuron survival  
(% of Control at 7 DIV)

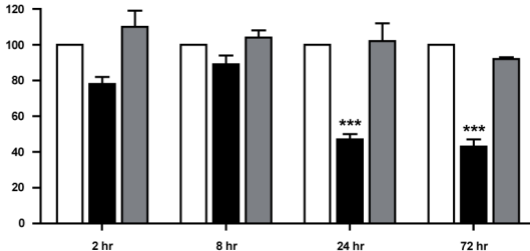

Supplement: Supplementary Figure 2 — Time-course of motoneuron survival in primary spinal cord cultures exposed to ACM-SOD1G93A. Primary wild-type (WT) rat spinal cord cultures (4 DIV) were exposed for 2, 8, 24, and 72 h to ACM derived from transgenic mice overexpressing SOD1G93A (ACM-SOD1G93A) and fixed immediately afterwards. Cell survival was assayed with immunocytochemistry. Graph showing the percentage of motoneurons that survived after treatment with ACMSOD1G93A, relative to control medium and with media derived from transgenic mice carrying the non-pathological human wild-type SOD1 gene (ACM-SOD1WT). Survival is shown relative to cultures treated with control media. Values represent mean ± s.e.m. from at least 3 independent experiments performed in duplicate, analyzed by One-Way ANOVA followed by a Tukey post-hoc test. ***P < 0.001 relative to control medium at 7 DIV. [file Presentation2.PDF]

**A**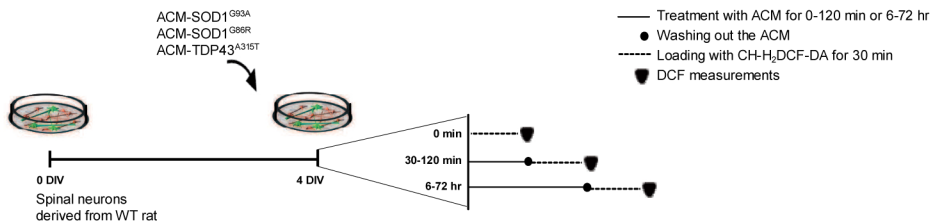**B**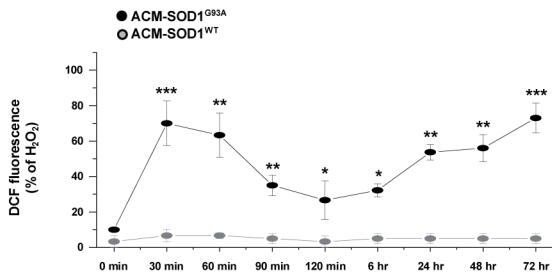**C**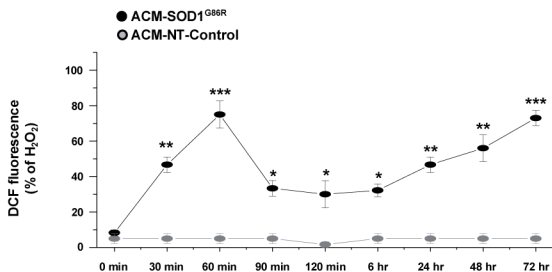**D**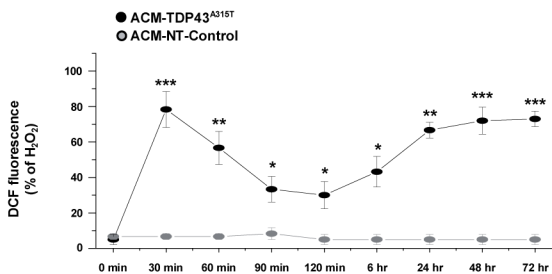

Supplement: Supplementary Figure 3 — Time-course of DCF fluorescence in spinal cord cultures exposed to ACM-SOD1G93A, ACM-SOD1G86R, or ACM-TDP43A315T. (A) Flow diagram of experiment. Primary wild-type spinal cultures (4 DIV) were exposed for 0, 30, 60, 90, and 120 min and 6, 24, 48, and 72 h with the different ACMs (solid lines), washed to remove the ACMs (filled circles), and loaded for 30 min with the fluorescent membrane permeable ROS/RNS probe CM-H2DCF-DA (dotted lines). Next, cultures were washed and DCF measurements were immediately performed (filled triangles). (B–D) Graphs showing the percentage of DCF fluorescent cells (including both motoneurons and interneurons) in cultures exposed to ACM-SOD1G93A (B), ACM-SOD1G86R (C), or ACM-TDP43A315T (D). Results obtained with different controls are included in the graphs: ACMSOD1WT (B), ACM-NT-Control from SOD1G86R−/−astrocytes (C), and ACM-NT-Control from TDP43A314T−/− astrocytes (D). DCF fluorescence is relative to cultures treated with H2O2, 200 μ M for 20 min (100%). Note that application of toxic ACMs resulted in an initial peak of DCF fluorescence at 30–60 min that was followed by a decline in the DCF signal which then started to steadily increase from 24 h on. Values represent mean ± s.e.m. from at least 3 independent experiments performed in duplicate, analyzed by t-test. *p < 0.05, **p < 0.01, ***p < 0.001 vs. control. [file Presentation3.PDF]

**A**

Application of:

 $\text{H}_2\text{O}_2$  $\text{H}_2\text{O}_2$  + Antioxidants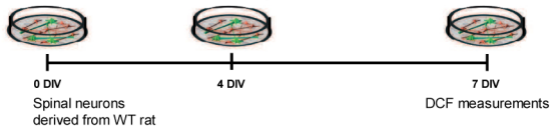**B**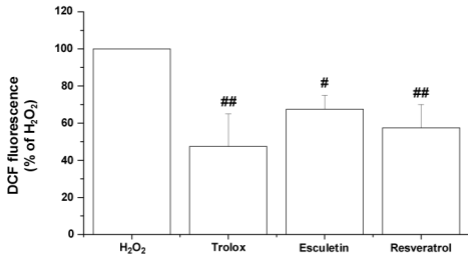

Supplement: Supplementary Figure 4 — Anti-oxidant resveratrol is as effective as Trolox and esculetin in reducing DCF fluorescence induced by H2O2. (A) Flow diagram of experiment. Primary spinal cord cultures (4 DIV) were exposed to 200 μ M H2O2 for 20 min in the presence or absence of anti-oxidants. Cells were washed and then loaded with the membrane permeable ROS/RNS probe CM-H2DCF-DA for 30 min. After washing, DCF fluorescence was measured immediately. (B) Graphs showing the percentage of DCF fluorescent cells (including motoneurons and interneurons) in cultures exposed to H2O2 and Trolox (1 μ M), esculetin (25 μ M), or resveratrol (1 μ M). Values represent means ± s.e.m. from at least 3 independent experiments, analyzed by One-Way ANOVA followed by a Tukey post-hoc test. ##P < 0.01, ###P < 0.001 relative to DCF fluorescence in H2O2 treated cultures. [file Presentation4.PDF]
