# Supplementary material for: Astrocytes expressing mutant SOD1 and TDP43 trigger motoneuron death that is mediated via sodium channels and nitroxidative stress
Source: Front Cell Neurosci. 2014 Feb 7;8:24. doi: 10.3389/fncel.2014.00024 (PMC3916762; doi:10.3389/fncel.2014.00024)
Supplement: Supplementary Table 1 — Effects of a wide range of concentrations of antioxidants on the survival of control motoneurons and those exposed to ACM-SOD1G93A. (A) Analysis of survival of motoneurons at 7 DIV in primary cultures after incubation for 4 days with multiple doses of the anti-oxidants Trolox, esculetin, and resveratrol. (B) Analysis of surviving motoneurons at 7 DIV in primary cultures after incubation for 4 days with ACM-hSOD1G93A alone (no drug), or with ACM plus different doses of the anti-oxidants. In bold are indicated concentrations at which concentration the anti-oxidant was selected for further analysis on preventing the toxicity induced by the diverse ACMs; those results are displayed in Figure 3. Values represent mean ± s.e.m. from at least 3 independent experiments performed in duplicate, analyzed by t-test vs. control. [file DataSheet1.PDF]

A

Application	Concentration	Motoneuron Survival (%)	Statistics (Control)
Control	-	100 ± 4	-
Trolox	0,1 µM	98 ± 29	0.9488
	0,25 µM	96 ± 40	0.9255
	0,5 µM	101 ± 1	0.8203
	0,75 µM	100 ± 4	1.000
	**1 µM**	**101 ± 1**	**0.8203**
	2,5 µM	95 ± 6	0.5262
	10 µM	93 ± 12	0.6095
	25 µM	89 ± 1	0.0559
	50 µM	85 ± 8	0.1688
	75 µM	82 ± 4	0.0335
	100 µM	83 ± 15	0.3350
Esculetin	2 µM	103 ± 2	0.5391
	10 µM	99 ± 1	0.8203
	15 µM	100 ± 1	1.0000
	20 µM	101 ± 10	0.9305
	**25 µM**	**100 ± 1**	**1.0000**
	30 µM	89 ±15	0.5177
	40 µM	71 ± 1	0.0022
	50 µM	58 ± 2	0.0007
Resveratrol	0,1 µM	101 ± 7	0.9073
	0,25 µM	102 ± 8	0.8340
	0,75 µM	100 ± 34	1.0000
	**1 µM**	**100 ± 1**	**1.0000**
	10 µM	91 ± 8	0.3712
	25 µM	88 ± 5	0.1342
	35 µM	85 ± 7	0.1363
	50 µM	73 ± 1	0.0028
	75 µM	66 ± 8	0.0191
	100 µM	55 ± 3	0.0008

B

Application	Concentration	Motoneuron Survival (%)	P-values (Control)	P-values (ACM-SOD1^{G93A})
Control	-	100 ± 3	-	0.0028
ACM-SOD1^{G93A}	-	56 ± 6	0.0028	-
Trolox	0,1 M	60 ± 14	0.0491	0.8058
	0,5 µM	79 ± 19	0.3363	0.3126
	**1 µM**	**84 ± 2**	**0.0144**	**0.0114**
	2,5 µM	78 ± 3	0.0066	0.0305
	10 µM	66 ± 6	0.0071	0.3039
Esculetin	**25 µM**	**85 ± 2**	**0.0141**	**0.0101**
Resveratrol	0,1 µM	60 ± 14	0.0491	0.8085
	0,25 µM	61 ± 16	0.0747	0.7844
	**1 µM**	**61 ± 6**	**0.0044**	**0.5874**
	10 µM	58 ± 8	0.0080	0.8512

Supplementary Table 1
Rojas et al., 2013
